# Optical Coherence Tomography Angiography: Revolutionizing Clinical Diagnostics and Treatment in Central Nervous System Disease

**DOI:** 10.14336/AD.2024.0112

**Published:** 2024-01-12

**Authors:** Zeqi Shen, Sheng Zhang, Weitao Yu, Mengmeng Yue, Chaoyang Hong

**Affiliations:** ^1^Postgraduate training base Alliance of Wenzhou Medical University (Affiliated People’s Hospital), Hangzhou, Zhejiang, China.; ^2^Center for Rehabilitation Medicine, Department of Neurology, Zhejiang Provincial People’s Hospital (Affiliated People’s Hospital), Hangzhou Medical College, Hangzhou, Zhejiang, China.; ^3^The Second School of Clinical Medicine, Hangzhou Normal University, Hangzhou, Zhejiang, China.; ^4^Center for Rehabilitation Medicine, Department of Ophthalmology, Zhejiang Provincial People’s Hospital (Affiliated People’s Hospital), Hangzhou Medical College, Hangzhou, Zhejiang, China.

**Keywords:** OCTA, OCT, fundus photography, CNS, neuro-ophthalmology

## Abstract

Optical coherence tomography angiography (OCTA), as a new generation of non-invasive and efficient fundus imaging technology, can provide non-invasive assessment of vascular lesions in the retina and choroid. In terms of anatomy and development, the retina is referred to as an extension of the central nervous system (CNS). CNS diseases are closely related to changes in fundus structure and blood vessels, and direct visualization of fundus structure and blood vessels provides an effective "window" for CNS research. This has important practical significance for identifying the characteristic changes of various CNS diseases on OCTA in the future, and plays a key role in promoting early screening, diagnosis, and monitoring of disease progression in CNS diseases. This article reviews relevant fundus studies by comparing and summarizing the unique advantages and existing limitations of OCTA in various CNS disease patients, in order to demonstrate the clinical significance of OCTA in the diagnosis and treatment of CNS diseases.

## 1. Introduction

Optical coherence tomography angiography (OCTA) is the latest generation of fundus imaging technology that quantitatively evaluates the blood vessels in the retina and choroid. The US Food and Drug Administration (FDA) approved the use of OCTA in 2015. Compared with traditional retinal imaging modes, OCTA has advantages such as being non-invasive, efficient, convenient, and inexpensive, making it widely used by ophthalmologists in clinical and research fields. Meanwhile, there has been much discussion about whether OCTA can also be used in neuro-ophthalmology and neurology. The retinal vascular network and cerebral vascular network exhibit astonishing similarities. The retina and brain share similar embryonic origins, anatomical features, and physiological features, and are considered extensions of the central nervous system (CNS). The pathological and physiological changes of retinal structure as well as the blood vessels can serve as a unique "window" to reflect the pathological changes in neurological diseases. As for the microvascular system, during embryonic development, the retina and CNS have similar mechanisms of angiogenesis, and their blood supply and vascular regulation processes are also closely related. Therefore, the homology between the retina and cerebral blood vessels forms the basis for studying potential biomarkers of cerebral microvascular status in cerebrovascular diseases. In addition, the retina and optic nerve are extensions of the diencephalon, and the unmyelinated axons of retinal ganglion cells form the retinal nerve fiber layer (RNFL), which is considered a projection of the CNS.

Based on the above, research into the changes in the fundus reflecting early vasculopathy in the CNS has increased considerably in recent years. To date, several fundus vascular visualization techniques have been applied to validate the association between fundus changes and CNS disease, including fluorescein angiography (FA), fundus photography, optical coherence tomography (OCT), and OCTA. Due to the time-consuming and invasive nature of FA, which can also cause severe allergic reactions in some patients, many previous studies have focused on fundus photography and OCT technology. Fundus photography, although simple and user-friendly, provides only a two-dimensional plane image of the fundus, limiting the depth observation and analysis of retina structure. With the advanced of imaging technique, OCT can reveal the three-dimensional structure of the retina in real time through longitudinal section tomography, which can more accurately help doctors locate the lesions and observe the morphology of retinal lesions. OCTA, as the latest non-invasive fundus vascular visualization technology, can further quantitatively assess the changes of fundus microvascular structure and function compared with OCT, enabling clinicians to objectively understand the vascular density and blood perfusion of the retina.

**Figure 1.**
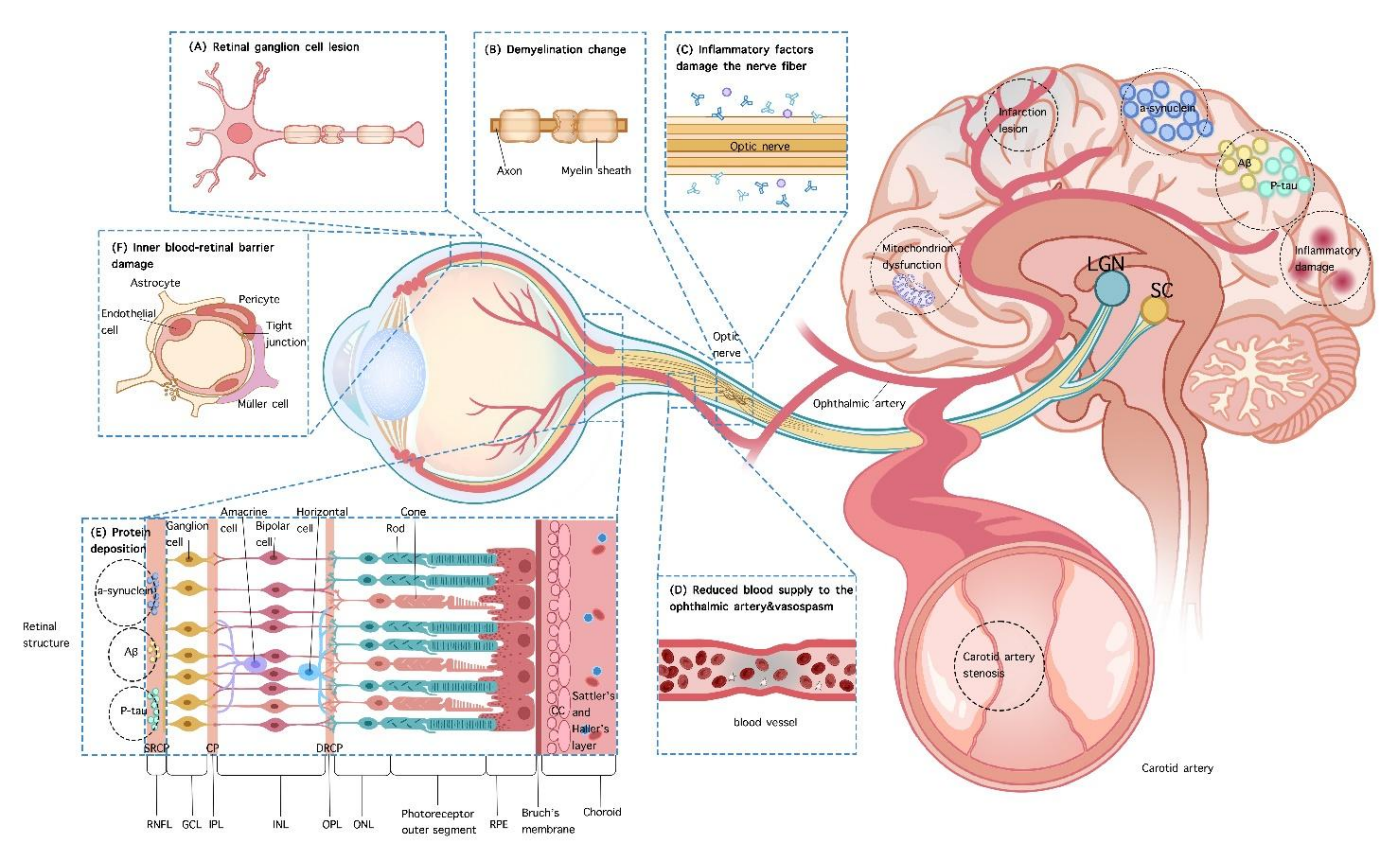
**The mechanisms that link fundus lesions and CNS diseases**. The primary targets of eye-brain comorbidities are the blood-retinal barrier, optic nerve, and retinal blood vessels. We classify CNS diseases into four categories and will describe how the above targets are compromised in each category of CNS diseases. (1) Neurodegenerative diseases, such as AD, PD, HD, and ALS can result in the damage of neurons and their corresponding nerve conduction pathways. Any damage to the visual pathway can lead to secondary retrograde or anterograde synaptic degeneration, resulting in the damage of retinal ganglion cells (A) and demyelination of the optic nerve (B); Amyloid-like proteins and collagen produced by certain diseases can also deposit in the retina, leading to damage to retinal microvasculature (E); (2) Neurological immune diseases, such as MS and NMOSD, the immune response disrupts not only the blood-brain barrier and causes demyelination of brain tissue, but also disrupts the integrity of the optic nerve myelin sheath (B) and blood-retinal barrier (F). In addition, antibodies and various inflammatory factors can directly damage the optic nerve, leading to the onset of optic neuritis (C); (3) Cerebrovascular disease, such as stroke and stenosis of the intracranial and extracranial arteries, can result in regional brain injury, decreased cerebral perfusion, impaired microvascular circulation, and ultimately insufficient retinal blood supply. For example, CAS can lead to a decrease in blood supply to the internal carotid artery, resulting in inadequate blood flow to the ophthalmic artery. The mechanism of migraine is different from the aforementioned cerebrovascular diseases. Migraine attacks can lead to retrobulbar vasoconstriction, which affects the blood flow and perfusion of retinal microvasculature (D); (4) CNS hereditary diseases. For example, WS is characterized by endoplasmic reticulum and mitochondrial dysfunction, resulting in neuronal apoptosis. Additionally, abnormal WS protein expression on the unmyelinated optic nerve can lead to optic nerve injury (B). CNS, central nervous system; AD, Alzheimer's disease; PD, Parkinson's disease; HD, Huntington's disease; ALD, amyotrophic lateral sclerosis; MS, multiple sclerosis; NMOSD, neuromyelitis optica spectrum disease; CAS, carotid artery stenosis; RNFL, retinal nerve fiber layer; GCL, ganglion cell layer; IPL, inner plexiform layer; INL, inner nuclear layer; OPL, outer plexiform layer; ONL, outer nuclear layer; RPE, retinal pigment epithelial; CC, choriocapillaris; SRCP, superficial retinal capillary plexus; ICP, intermediate capillary plexus; DRCP, deep retinal capillary plexus; LGN, lateral geniculate nucleus; SC, suprachiasmatic nucleus.

A large number of studies have shown that OCTA technology may provide a new perspective for the neural mechanisms and subclinical pathology research of CNS diseases, and its potential is particularly worth looking forward to and continuously exploring. The potential reasons why CNS diseases affect the structure of the fundus and vascular system may include the following: (1) Vasogenic lesions. Some CNS diseases, such as carotid artery stenosis, stroke, cerebral infarction, and migraine, not only reduce cerebral blood supply but also affect ocular blood flow perfusion. Some other CNS diseases, such as Alzheimer's and Parkinson's, can deposit proteins and collagen in the ocular blood vessels, especially the superficial retinal plexus, thereby affecting retinal and choroidal blood flow perfusion; (2) Degenerative changes. The axons of retinal ganglion cells form the optic nerve, which extends to the lateral geniculate nucleus of the thalamus and the superior colliculus of the midbrain, allowing us to see images. The aggregation of axons in many ganglion cells ultimately forms the optic nerve. Like other CNS axonal injuries, damage to any part of the optic nerve pathway can cause retrograde and anterograde degeneration. Subsequently, damage to neural pathways can lead to a neurotoxic environment, including oxidative stress, deprivation of neurotrophic factors, excitatory toxicity levels of neurotransmitters, and abnormal aggregation of proteins and debris. This secondary pathologic change can further damage the fundus of the eye; (3) Immunogenic damage. Some autoimmune CNS diseases, such as multiple sclerosis and optic neuromyelitis spectrum diseases, together produce multiple inflammatory factors and cause damage to the retina, affecting inner-blood retina barrier and optic nerve; (4) Mitochondrial dysfunction. Some CNS disease such as Huntington's disease, Leber’s hereditary optic neuropathy and Wolfram syndrome can affect the patient's mitochondria, and mitochondrial dysfunction then leads to a lack of energy supply to the cellular axons, which in turn leads to further ganglion cell damage. Figure 1 shows that CNS diseases affect the structure and blood flow of the retina and choroid through different mechanisms.

In summary, our review is based on the development of fundus imaging technology and focuses on the application of fundus photography, OCT, and OCTA technology in the clinical diagnosis and treatment of CNS disease. In addition, we discuss the clinical importance of OCTA parameters as possible markers for early identification and continuous monitoring of CNS lesions.

## 2. Materials and methods

We conducted a comprehensive review of the current literature up to December 2023 using Google Scholar, PubMed, Mendeley search engines. The terms “optical coherence tomography”, “optical coherence tomography angiography”, “neuro-ophthalmology”, “central nervous system” and “neurology” were typed in the aforementioned search engine tools in order to identify publications that are related to the use of OCTA. Other associated terms were also used including Alzheimer's disease (AD), Parkinson's disease (PD), Huntington's disease (HD), multiple sclerosis (MS), neuromyelitis optica spectrum disease (NMOSD), stroke, cerebral infarction, migraine, carotid artery stenosis (CAS), Leber’s hereditary optic neuropathy (LHON), amyotrophic lateral sclerosis (ALS), Wolfram syndrome (WS). We used approximately 150 relevant manuscripts published from 2015 to December 2023 as references, focusing on specific descriptions of OCTA and its application in neurological and neuro-ophthalmic diseases. Our focus on publications from 2015 onwards is due to the approval of OCTA by the US FDA in that year.

## 3. Basic Principles and Algorithms in OCTA

Different OCTA devices on the market use several algorithms to obtain images of the retinal vascular system. Currently, widely utilized OCTA devices are listed as follows: Topcon DRI Triton (DRI-OCT Atlantis OCT, Topcon Corporation, Japan), AngioVue device (RTVue XR Avanti with AngioVue, OptovueInc, Fremont, California, USA), Zeiss PLEX Elite 9000 (Zeiss PLEX Elite 9000; Carl Zeiss Meditec, Dublin, California, USA), Zeiss Cirrus HD 5000 (CIRRUS™ HD-OCT Model 5000 instrument, Carl Zeiss Meditec, Dublin, California, USA), Heidelberg Spectralis (Heidelberg Spectralis System, Heidelberg Engineering, Heidelberg, Germany), Heidelberg Spectralis II (Heidelberg Spectralis II System, Heidelberg Engineering, Heidelberg, Germany), Nidek RS-3000 Advance 2 (RS-3000 Advance 2 OCT, NIDEK Corporation Ltd., Gamagori, Japan), Spectralis HRA+OCT (Heidelberg Engineering, Heidelberg, Germany) and VG 200 (SVision Imaging Limited, Luoyang, China).

**Figure 2.**
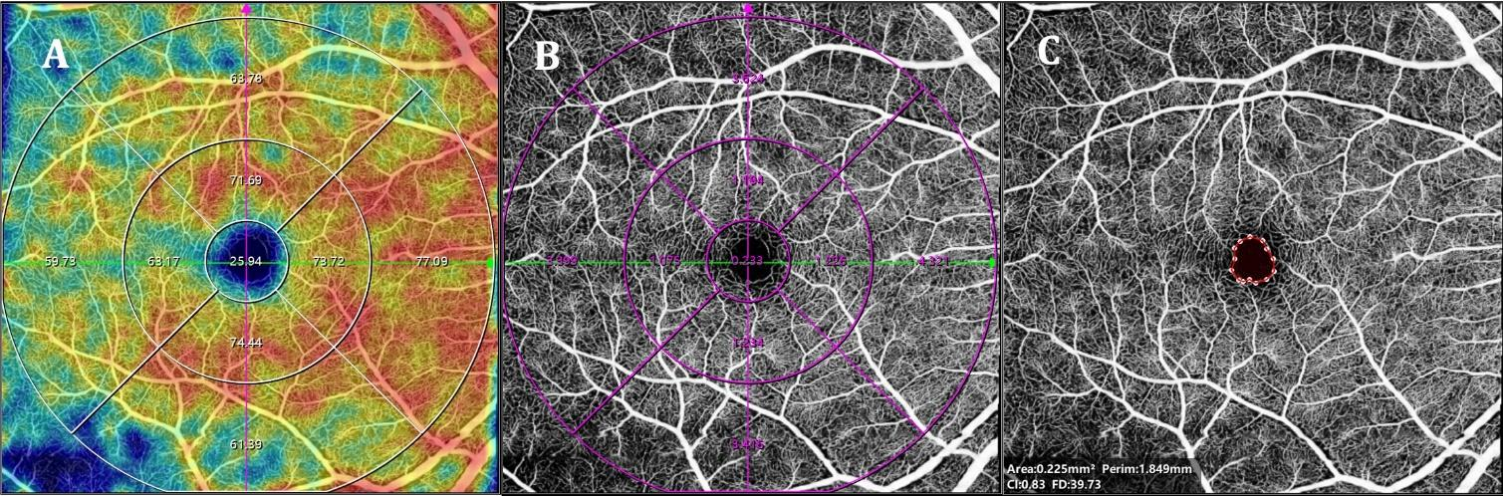
**Examples of retina parameters measured by OCTA (VG200S, SVision Imaging, Ltd., Henan, China)-equipped software (vangogh v3.0.188)**. OCTA can automatically measure and obtain various retinal blood flow related parameters such as vessel density (A), perfusion area (B) and FAZ (C). OCTA, optical coherence tomography angiography; FAZ, fovea avascular zone.

OCTA imaging is based on the same principles as conventional OCT, and it identifies the different tissues of the retina and choroid based on reflectance differences: retinal nerve fiber layer (RNFL), ganglion cell layer (GCL), inner plexiform layer (IPL), inner nuclear layer (INL), outer plexiform layer (OPL), outer nuclear layer (ONL), external limiting membrane (ELM), photo-receptor layer, retinal pigment epithelial (RPE), choriocapillaris (CC), Sattler’s layer and Haller’s layer. In addition, OCTA allows the acquisition of images of specific retinal vascular networks by segmentation. Most existing OCTAs utilize this principle to divide the retina and choroid into six main different regions, including radial peripapillary capillary plexus (RPC), superficial retinal capillary plexus (SRCP), intermediate capillary plexus (ICP), deep retinal capillary plexus (DRCP), CC and choroid (Sattler’s layer and Haller’s layer). There are still relatively few studies using OCTA to study the CC and choroidal vessels in CNS diseases patients. The main reason for this is the inability of existing OCTA techniques to accurately segment the deep choroidal vascular layer and accurately measure its vascular parameters. Using OCTA-equipped software, researchers can obtain quantitive data of the above each layer, in order to evaluate the presence of new blood vessels, loss of capillary perfusion, increased vascular curvature, central foveal avascular zone (FAZ), parafoveal area and areas of foveal non-perfusion. An example of parameters measured in retina layer using OCTA-equipped software is shown in Figure 2.

The quality of OCTA scanning involves two aspects: resolution and scanning range. It is known that the scanning field of view expands with the decrease of resolution. However, with the development of OCTA technology, the latest OCTA can balance high resolution and wide scanning range, which is swept source OCTA (SS-OCTA). The longer wavelength and faster imaging speed of SS-OCTA facilitate better visualization of structure and vasculature below pigmented tissue with a larger field of view of the posterior segment. To date, the largest field of view obtained with a single capture is 29 × 24 mm with an A-scan rate of 400 kHz. However, the maximum scanning range currently used in CNS diseases research is mostly 6 × 6mm. The reasons for not adopting a wider scanning mode include: (1) The technology of OCTA ultra-wide-angle scanning is too new to be applied in CNS research; (2) CNS diseases patients are mostly elderly who were difficult to cooperate with ultra-wide-angle scanning, as the quality of the scanned image is prone to artifacts.

## 4. Neurodegenerative Disease

### 4.1 AD

AD is a prevalent neurodegenerative disorder that results in significant cognitive decline and is the leading cause of dementia among older individuals. Despite ongoing research, the precise etiology of AD remains uncertain. However, AD is primarily associated with the excessive accumulation of Aβ amyloid plaques in the extracellular space, neuronal loss, the presence of neurofibrillary tangles within neurons, and abnormal intracellular tau protein. Previous investigations have indicated that Aβ amyloid can also accumulate in the retina of individuals with AD, mirroring the pattern observed in the brain. Numerous studies have provided evidence of retinal vascular involvement in AD. Numerous studies have provided evidence indicating a correlation between retinal vascular alterations and cognitive declined, as observed through the utilization of fundus photography images obtained from deceased individuals [1, 2]. Scholars have postulated that within individuals affected by AD, the accumulation of Aβ amyloid in the retina, in conjunction with the circulatory system, leads to impairment of the retinal microvascular system, ultimately resulting in structural and vascular impairment of the retina. Furthermore, ocular manifestations that may manifest in AD patients encompass abnormal pupillary response, diminished contrast sensitivity, as well as thinning of GCL and RNFL, peripapillary atrophy, and retinal thinning.

The present diagnostic approaches for AD involve invasive procedures such as cerebrospinal fluid (CSF) and blood analyses, as well as costly PET scan imaging. Consequently, the prioritization of cost-effective, noninvasive tests for early detection of AD is of utmost importance. OCTA is a rapidly developing technique which generates angiographic images non-invasively, allowing the retinal microvasculature to be visualized at various retinal depths and quantify the vascular parameters within a certain range. This undoubtedly provides a way to a safer, easier, and faster diagnosis of AD. Previous research on AD has focused on the three contiguous phases of AD: (1) Previous family history of AD (FH+) or carried at least one ApoE e4 allele. The exact role of family history and the ApoE gene in the pathogenesis of AD is still under investigation. According to current research, ApoE is the strongest risk gene for sporadic Alzheimer's disease [3, 4]; (2) Preclinical AD. Patients who have not yet demonstrated clinical signs or symptoms of AD, but for whom pathologic biomarkers of AD have been identified as positive; (3) AD with cognitive impairment. This section will provide an overview of the association between AD and fundus vascular changes observed by OCTA based on the above-categorized disease course.

### 4.1.1 Family history of AD (FH+) and ApoE gene.

Previous studies using OCTA in populations of FH+ and carrying the ApoE gene are relatively few, and the conclusions are controversial. Past studies have found significant reductions in retinal thickness and retina vascular parameters in ApoE positive patients compared to gene-negative control group. In particular, SRCP vessel density (VD) and perfusion density (PFD) were significantly reduced [5, 6], as was DRCP VD [5, 7]. In addition, people with a FH+ and those who carry the ApoE gene also have significantly reduced RPC VD and capillary flux index compared to healthy controls (HC) [6, 7]. However, a study by López-Cuenca et al. showed that choroidal thickness and FAZ, on the other hand, did not change significantly in patients with a FH+ and a positive ApoE gene compared to HC [8]. This may be because the ApoE gene and family history of AD, as the high-risk factors for the development of AD, do not widely involve the fundus. However, they have some degree of effect on some of the retinal vascular parameters.

### 4.1.2 Preclinical AD

In preclinical AD patients, AD pathophysiologic changes have occurred in the brain, but the associated AD symptoms have not yet become evident. Preclinical AD can be diagnosed by PET or quantitative measurement of Aβ amyloid and Tau protein in CSF obtained by lumbar puncture or in blood samples.

A study by O'Bryhim et al. showed that OCTA images of preclinical AD patients had a significantly reduced inner foveal thickness and enlarged FAZ area compared to HC [9], which is consistent with the fundus manifestations in patients with a previous diagnosis of AD who already had symptoms of dementia [10, 11]. This also represents the fact that in the early stage of AD, the retinal vascular may have begun to undergo apoptosis and vascular attenuation due to the deposition of Aβ amyloid and collagen. In contrast, the study by van de Kreeke et al. yielded different results. He found that retinal VD (inner and outer ring of the macula and around the optic nerve head (ONH)) was higher in individuals with preclinical AD. Moreover, retinal VD was significantly correlated with the Aβ protein load score [12]. In conclusion, this may be due to an inflammatory response in the retina during the early stages of Aβ amyloid accumulation, which results in increased blood flow. However, the continued inflammatory response and the accumulation of Aβ amyloid then led to further retinal damage [13], impairment of the microvascular system, and ultimately a decrease in retinal VD.

### 4.1.3 AD and AD-induced cognitive impairment.

Compared with the previous two phases, there is a more abundant number of research on retinal changes in patients with AD and in patients with mild cognitive impairment (MCI) due to AD, although the findings are still controversial. Some studies have shown that the fundus of AD patients are not significantly different from the HC [14, 15]. Nevertheless, more studies have found that the thickness of multiple retinal layers is significantly lower in AD patients compared to HC, including whole retinal thickness [16-20], ganglion cell plexiform layer (GC-IPL) [1, 16, 19, 21, 22], ganglion cell complex (GCC) [23], outer retinal layer [24] and RNFL [16, 17, 19, 23, 25-29]. There was also a significant reduction in choroidal thickness [10, 11, 30-34]. Using OCTA, some studies further confirmed these results. In addition, the researchers found a positive correlation between the degree of fundus damage and AD severity that macular thickness and RNFL were significantly thinner in patients with moderate to severe AD compared with those with mild AD [20]. It may indicate that retinal thickness can reflect AD progression. Moreover, retinal thickness was discovered to be associated with the degree of cognitive impairment (MMSE and MoCA scores) [19, 20, 23, 35], brain parenchymal volume [35] and the amount of Aβ amyloid and tau protein in the CSF [22] in AD patients.

**Figure 3.**
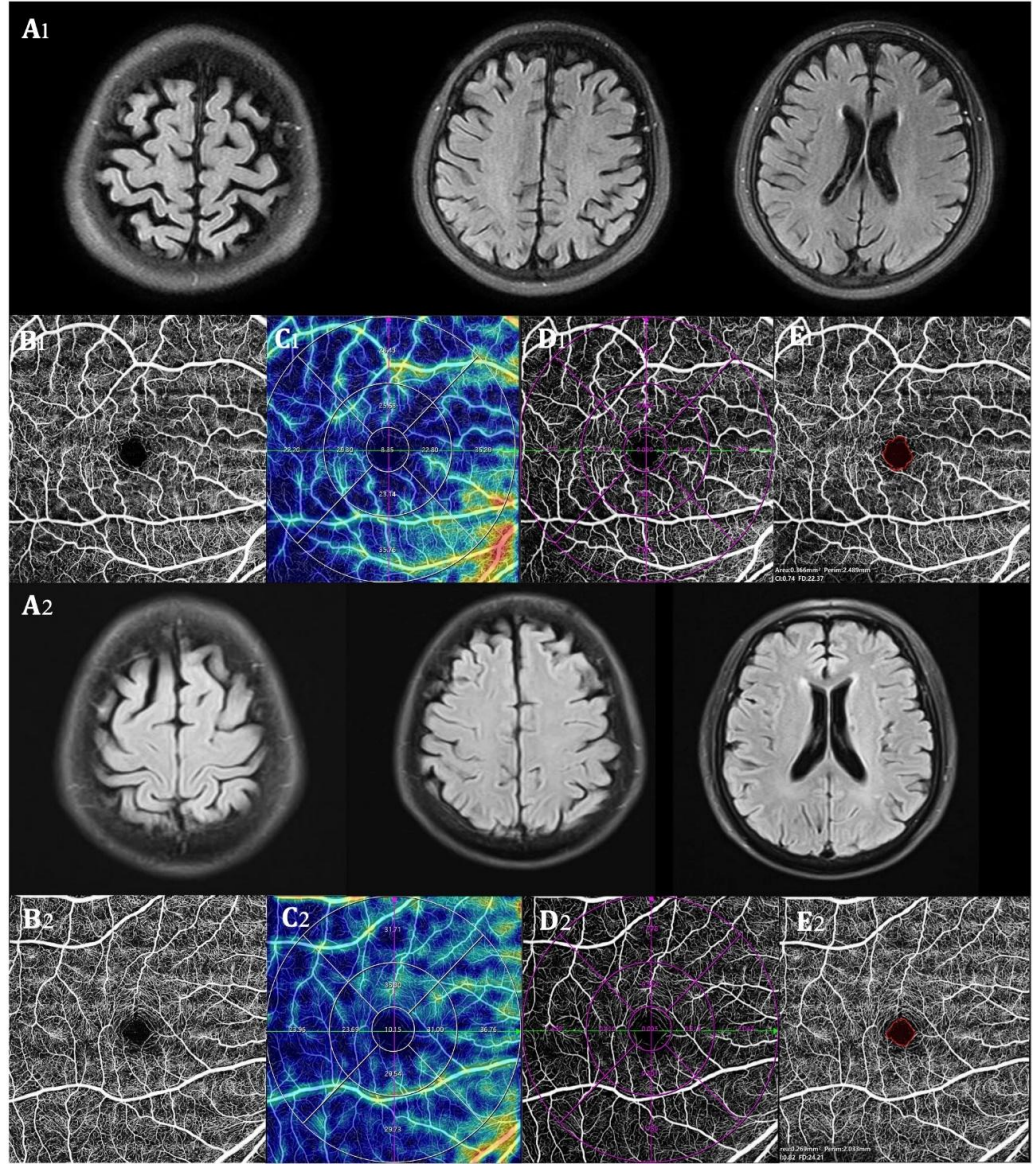
**Comparisons of OCTA parameters between an AD patient and a healthy control**. A 76-year-old female was admitted to the hospital due to “memory loss for one week”. The baseline brain MRI T2-FLAIR sequence showed an obvious cortical atrophy in her brain (A1). Genetic and CSF testing results supported AD diagnosis because she had ApoE e4 and A+T+. Compared with the healthy control (had no cognitive impairment and no brain atrophy on brain MRI T2-FLAIR, A2), the AD patient showed a decrease in retinal VD (B1 *vs.* B2), the SRCP VD (C1 *vs.* C2) and perfusion area (D1 *vs.* D2), but an increase in FAZ area (E1 *vs*. E2) on OCTA. A+: soluble Aβ 42/40 ratio decrease in CSF; T+: increase p-Tau181 protein content in CSF. MRI, magnetic resonance imaging; CSF, cerebrospinal fluid; AD, Alzheimer's disease; OCTA, optical coherence tomography angiography; SRCP, superficial retinal capillary plexus; VD, vessel density; FAZ, fovea avascular zone.

Using OCTA to further look at retinal and choroidal blood flow parameters in AD patients, the investigators found that retinal blood flow was significantly lower than in HC [10, 19]. Several blood flow parameters were significantly lower in SRCP [11, 18, 36-47] and DRCP [18, 29, 37, 39, 43, 44, 46, 47] compared to HC, such as VD, PFD, vessel length density (VLD), and fractal dimension (FD). Some studies also found significant reductions in blood flow parameters in the ICP [39, 43], RPC [18, 41], and CC [48]. FAZ area was lager in comparison to HC [10, 29, 37]. Researchers have also found that correlations between SRCP and disease duration [23], degree of cognitive impairment [23, 36, 42, 49], and brain parenchymal volume [50] in AD patients continue to manifest. Biscetti et al. found that the retinal FD is correlated with the CSF Aβ42/t-tau ratio [43]. An example of one AD patient examined by OCTA is shown in Figure 3.

It is noteworthy that SRCP is the most significantly altered vascular parameter repeatedly demonstrated at this stage of AD compared with other stages [11, 18, 36-47]. Significant alterations in SRCP are likely to be related to the pathogenesis of AD. Aβ amyloid is more likely to accumulate in the inner layer of the retina, near the GCC [51], and SRCP is located in the RNFL and GCC. Therefore, we hypothesize that SRCP is the primary tissue attacked by Aβ amyloid accumulation. As the disease progresses, Aβ amyloid accumulates in all layers of the retina, and will trigger deep retinal vascular and even damage choroidal layer.

In summary, the effects of AD on the retina are comprehensive and widespread. Both retinal and choroidal thickness and vascular parameters are affected to varying degrees. However, whether specific changes occur in the retina and choroid of AD patients remains to be further explored by researchers. In a recent 2023 study using OCTA to observe the effects of dementia on the retina, patients with AD-related dementia had thicker RNFL and GCC in the nasal sector and higher FD in SRCP and DRCP than other dementia populations, but had significantly lower VD in the CC [23]. It is likely to represent that the effects of AD on the retina are somewhat specific. In another study comparing early-onset AD with late-onset AD, Robbins et al. did not find significant differences in the retinas of different types of AD patients, but still confirmed that the retinal VD and PFD were significantly lower in AD patients compared to HC [52]. Mirzania et al. found that male AD patients had significantly lower retinal VD and PFD compared to female AD patients [53].

### 4.2 PD

PD is a growing global health problem with increasing prevalence due to an aging population and is the second most common neurodegenerative disease after AD. It has the fastest-growing disability, prevalence, and mortality rates [54]. The pathological hallmarks of PD are loss of dopaminergic neurons in the substantia nigra pars compacta and accumulation of misfolded a-synuclein, followed by dopaminergic neurodegeneration[55, 56]. Dopamine, as a neurotransmitter for visual signal processing in the retina, also has a critical role in the retina. Dopamine deficiency in PD patients can lead to a range of visual symptoms, including vision loss, hallucinations, and visuospatial orientation deficits. Sometimes, patients' visual symptoms even precede motor symptoms. In the retina of autopsied subjects with PD, researchers found the presence of phosphorylated a-synuclein. PD has likely led to changes in the structure and function of the retina. Animal models of PD have also shown that a-synuclein is deposited on arterioles in the superficial layers of the retina [57, 58]. Therefore, the researchers hypothesized that changes in the retinal vascular system of PD patients could be used as a biological marker for screening, diagnosing PD, and inferring disease severity and progression.

Previous OCTA studies found significantly reduced thickness of multiple retinal and choroidal layers in PD patients compared to HC, including total retinal volume and thickness [59, 60], outer retinal layer [61], GCC [62], GCL [60, 63], GC-IPL [59], INL [64], RNFL [59, 60, 63-66], and choroid[61]. In summary, PD presents a wide range of effects on retinal and choroidal thickness. In studies using OCTA to observe retinal and choroidal vascular parameters in patients with PD, several parameters such as VD, PFD, VLD, and FD were significantly lower in SRCP and DRCP compared with HC [66-68]. Some studies have also found significant reductions in RPC [64, 65, 69, 70] and choroidal [61, 71] vascular parameters. Choroidal blood flow parameters include choroidal vascular index (CVI) and choroidal vascular volume (CVV), which measure blood flow in the large and medium-sized choroidal vessels (Haller's layer and Sattler's layer), and CC FD.

The Hoehn-Yahr Scale (H-Y scale), the Unified PD Rating Scales-III scale (UPDRS-III scale), and the Cognitive Impairment Scale can be used to evaluate and monitor the severity and disease progression of PD. A negative correlation has been demonstrated between retinal vascular parameters in PD patients and the disease duration, and degree of disease progression (H-Y score, UPDRS-III score and MMSE score) [62, 64, 68, 72]. In addition, patients with mild PD exhibited significant differences in thinning RNFL thickness, reduced retinal VD, and significantly smaller FAZ area compared to patients with moderate to severe PD [64, 72, 73]. This suggests that retinal thickness as well as vascular parameters may also reflect disease progression in PD patients.

**Figure 4.**
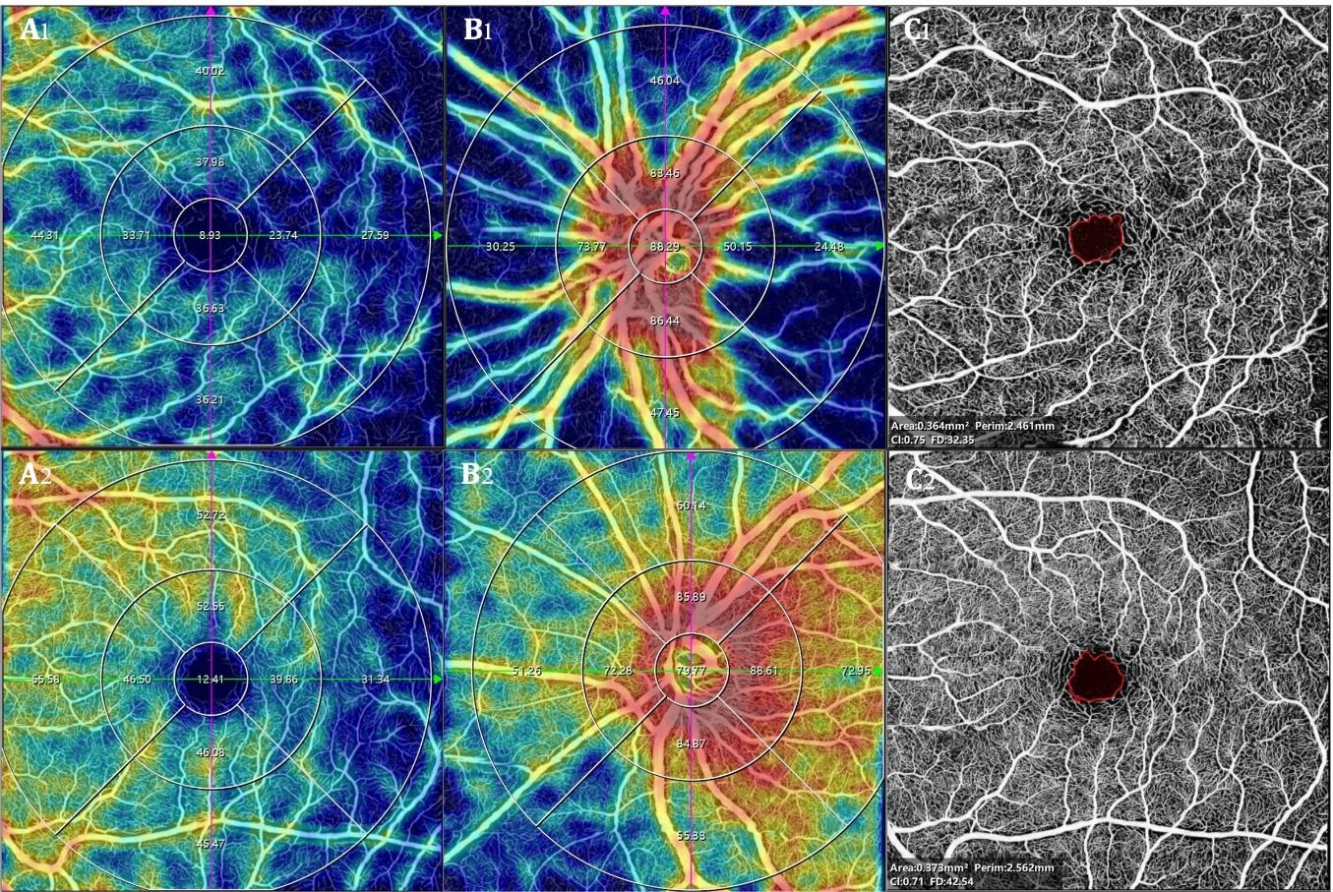
**Comparisons of OCTA parameters between a PD patient and a healthy control**. A 68-year-old female patient was admitted to the hospital due to “tremors in the right hand and lower limbs for 5 years and worsened for 2 months”. The patient showed positive for Medobar test and so was diagnosed as PD. Compared with the healthy control who had the same age and gender, the PD patient showed a decrease in SRCP VD in macular (A1 *vs*. A2) and optic disc area (B1 *vs.* B2) and FAZ area (C1 *vs.* C2) on OCTA. PD, Parkinson's disease; OCTA, optical coherence tomography angiography; SRCP, superficial retinal capillary plexus; VD, vessel density; FAZ, fovea avascular zone.

In summary, several vascular parameters of the retina and choroid were significantly lower than HC, representing overall damage to the ocular microvascular system in PD patients, which is consistent with the disease mechanism of PD. Presumably, the FAZ area should increase in PD patients compared to the HC. However, previous studies using OCTA have yielded contradictory findings, with some researchers finding no significant change in FAZ area in PD patients [59, 60], while others found that FAZ area was significantly smaller in PD patients than in HC [69, 72, 74]. Furthermore, as the disease progressed, the FAZ area even decreased with increasing disease severity [72]. Additionally, the circularity of the FAZ was reduced compared to HC [59, 69]. Murueta-Goyena et al. [74]not only found that the FAZ area was smaller in PD patients compared to HC, but also that FD, PFD, vascular skeleton density (VSD), and vascular perimeter index (VPI), and lacunarity were increased in the macular fovea region in SRCP and DRCP compared to HC. The FAZ region is surrounded by a continuous network of capillaries in the retina, and the FAZ region contains dopamine neurons in the human retina [75, 76]. As PD progresses, dopamine neurons in the FAZ region are damaged, promoting the pathological proliferation of capillaries around the FAZ region, leading to an increase in vascular complexity and heterogeneity in the fovea of the macula, ultimately resulting in a decrease in the FAZ area and circularity. An example of one PD patient examined by OCTA is shown in Figure 4.

Results of PD studies are still contradictory, which may be due to: (1) the heterogeneity of results caused by the varied severity of PD; (2) the small sample size, and (3) the non-uniformity of OCTA machines used in different studies. In the future, it will be necessary to conduct homogeneous prospective multicenter studies with larger sample sizes in order to gain a more precise and in-depth understanding of the associations between fundus biomarkers measured by OCTA and PD disease.

### 4.3 HD

HD is an inherited neurodegenerative disorder characterized by progressive motor dysfunctions, cognitive impairments and personality changes. HD is caused by an expanded polyglutamine repeat encoded by a (CAG)n block in exon 1 within the IT15 gene encoding the protein huntingtin (htt) [77, 78].

Research on the fundus in HD patients remains limited. Previously, researchers observing the retina through fundus photography concluded that histological abnormalities in these patients' retinas were not significant. Even in autopsies of HD patients with advanced diseases (28 years of medical history), no histological abnormalities were found in the fundus. Microscopically, the entire HD retina showed no obvious histopathological changes. All retinal neurons exhibited nuclei without any HD characteristics of intranuclear inclusions, cytoplasmic aggregates, or nuclear indentations [79]. A study by Paulus et al. found electrophysiologic changes in 19 HD patients that were suggestive only of abnormal cone function [80].

With the development of fundus imaging techniques, Gatto et al. used OCT found significant thinning of the RNFL in the temporal and superior regions in HD patients [81]. However, no difference was found in the mean total RNFL thickness between HD patients and HC. The same results were found in a study by Kersten et al [82]. In addition, Kersten found a significant negative correlation between temporal RNFL thickness and HD disease duration, and a significant negative correlation between macular volume and disease duration as well as motor scores.

Gulmez Sevim et al. found that HD patients had a significant decrease in the thickness of the peripapillary RNFL (pRNFL), macular RNFL (mRNFL), GCL, IPL, INL, OPL and ONL in the temporal sector and a significant increase in the thickness of the outer retinal layer [83]. Of these, RNFL and GCL thicknesses were most strongly associated with HD, and were correlated with HD disease duration, burden score, CAG triplet repeat, total motor score on the Huntington's Disease Rating Scale (TMS-UHDRS score), and the Independence Scale were all correlated. Andrade et al. found that submacular choroidal thickness was significantly thinner in HD patients than in HC. Besides, no significant difference in retinal thickness was found between the two populations. However, mean, superior, inferior, and nasal macular thicknesses in HD patients were negatively correlated with TMS-UHDRS scores [84].

OCTA, the most up-to-date fundus imaging technique, has also been utilized by researchers to explore the retina of HD patients in greater depth. The latest OCTA studies have further confirmed the presence of fundus lesions in HD patients. Amini et al. found [85] that HD patients had significantly lower macular thickness in the inner superior, outer superior, and outer inferior regions and significantly lower pRNFL thickness in the temporal region compared to HC. Also, macular thickness in HD patients was negatively correlated with the duration of HD. However, the difference in retinal capillary density in HD patients was not statistically significant compared to HC. Di Maio et al. [86] reported statistically significant attenuation of the choroidal thickness in 16 HD patients, but no difference was found between HD and HC in SRCP and DRCP VD. These results show that the retinal structure of HD patients, especially the RNFL thickness, is more stable with significant differences compared to HC, and also shows a correlation with the severity and progression of HD. It may be due to the fact that (1) HD is a neurodegenerative disease, and previous drosophila and mouse models of HD exhibit severe neuropathology, including progressive disorganization of the retinal photoreceptor layer and retinal dysfunction. The above results might suggest that HD in the retina may exhibit the same neuronal pathology as in the brain [87-89]. The RNFL damage in HD patients may be due to the same neuronal damage occurring in the optic nerve. Impairment of the RNFL has also been commonly found in several previous studies in neurodegenerative diseases such as AD and PD; (2) HD is a genetic disorder that affects mitochondria. Previous studies have shown that mitochondrial dysfunction impaired mitochondrial transport function, leading to insufficient energy supply to small cell axons in mitochondrial diseases, resulting in the loss of temporal retinal ganglion cells and their axons [90, 91]. Impairment of the optic nerve, particularly of the RNFL in the temporal region, is a recognized feature of many mitochondrial diseases. For example, LHON, Friedreich ataxia and so on [92-95]. The thinning of RNFL in the temporal region of HD patients may also be related to mitochondrial dysfunction, and the damage of RNFL also causes secondary changes in retinal macular volume and thickness.

However, previous studies of retinal vascular biomarkers in HD patients have failed to find positive results. The retinal vascular parameters of HD patients are not significantly different from those of HC and are not closely related to the severity and duration of HD.

## 5 Central nervous system demyelinating disease

### 5.1 Vascular pathology in MS and NMOSD

MS [96, 97] and NMOSD [98] are idiopathic, inflammatory, and demyelinating disorders of the CNS, with lesions involving the brain, spinal cord, and optic nerves. While the clinical manifestations of MS and NMOSD diseases are very similar, the etiology of these two diseases differs. The pathogenesis of MS is not yet fully understood, but researchers believe that Epstein-Barr virus (EBV), sunshine (UVB), smoking and vitamin D, combined with an individual’s genetic background, play important roles in the causal pathway that results in MS development [99]. The characteristic pathological hallmark of MS is perivenular inflammatory lesions, leading to demyelinating plaques [100]. The inflammatory infiltrates contain T-lymphocytes, dominated by MHC class I restricted CD8+ T-cells. MS can lead to oligoclonal band positivity. Positive oligoclonal bands indicate the presence of humoral immune responses in CSF.

In many cases, NMOSD is characterized by the presence of the antibody of the water channel aquaporin-4 (AQP4-ab) in the astrocytic in CNS. AQP4-ab plays a major pathogenic role in the development of NMOSD and is also one of the most important pieces of evidence that helps physicians differentiate between NMOSD and MS [98, 101]. However, approximately 20% of NMOSD patients are AQP4-ab negative. Clinically, early differentiation between MS and NMOSD is crucial because they have different pathophysiology and treatments. Several drugs used in the treatment of MS are ineffective or even harmful in patients with NMOSD.

Previous studies have found that both MS and NMOSD can cause fundus changes, we may be able to use OCTA to differentiate between both MS and NMOSD. Based on the pathogenesis of MS and NMOSD, we hypothesized that MS and NMOSD cause changes in the fundus structure and microvascular system, most likely due to: (1) Primary vascular dysfunction. Both MS and NMOSD themselves trigger changes in retinal perfusion [102]; (2) Neural axonal decline in MS and NMOSD. The reason for the reduction in RNFL, GCC, GCL thickness, and even macular thickness may be because in human vision the first-order, second-order, and third-order neurons and their axons are hardwired projections of the human brain and transmit analog and digital signals [103]. Anatomically, GCC, GCL, and RNFL represent the first-order in the pathway. Irreversible axonal damage at any point in this pathway causes retrograde transsynaptic axonal degeneration, which inevitably leads to atrophy of the inner retinal layers (RNFL, GCC, and GC-IPL), or even the atrophy and thinning of the total retina thickness. It is possible that neuronal and axonal decline results in reduced metabolic activity of the inner retinal layers with consecutive lower oxygen and blood demand and regression of vessels. In turn, ischemia and hypoxia further lead to extensive damage to the retina and choroid; (3) Autoimmune response. Previous studies have suggested that meningeal inflammation may be the main driving factor for cortical demyelination in MS patients, and cortical demyelination may cause damage to the retinal structure of MS patients, leading to secondary damage to the retinal vascular system. In addition, MS can lead to an inflammatory response in the retina [104]. Previous autopsy studies have shown that most MS patients have retinal Müller cell hypertrophy and atrophy, as well as microglial cell activation in the eyes [105, 106]. These cells are crucial for the composition and integrity of the blood-retinal barrier (BRB). Damage to the BRB will inevitably further affect the blood supply to the retina. In patients with NMOSD, AQP4-ab plays a major pathogenic role in disease progression. Previous studies have shown that while wild-type mice rapidly cleared retinal swelling, AQP4-/- animals exhibited a sustainedly increased retinal thickness associated with retinal hyperperfusion, albumin extravasation, and upregulation of glial fibrillary acidic protein (GFAP). Therefore, the investigators hypothesized that genetic ablation of AQP4 leads to a functional derangement of the retinal gliovascular unit with retinal hyperperfusion during autoimmune CNS inflammation. This leads to structural damage to BRB and albumin extravasation. Ultimately, the retinal structure and vascular system are damaged. In addition, the autoimmune response has the potential to damage the BRB, leading to destruction of the retinal vascular system; (4) Decreased cerebral perfusion in MS and NMOSD. Previous studies have shown significant reductions in cerebral gray and white matter blood flow in both relapsing-remitting [107-109] and primary progressive [110, 111] forms of MS patients. Researchers have suggested that reduced cerebral perfusion in MS patients may be due to diffuse perivascular inflammation. The inflammatory response leads to microvascular damage, thrombosis, and fibrin deposition. Additionally, MS patients may also have obstructed outflow from extracranial veins, a condition known as chronic cerebrospinal venous insufficiency (CCSVI). A combination of factors culminates in damage to the gray and white matter of the brain. The gray matter and white matter of NMOSD patients were also significantly reduced [112]. Moreover, the more episodes of optic neuritis (ON), the lower the gray matter perfusion in the brain. The decrease in cerebral perfusion can lead to a lack of blood supply to the retina; (5) Episodes of ON. ON is a common feature in patients with MS and NMOSD and is one of the core symptoms of both diseases. ON is the presenting symptom of MS in 25% of cases and occurs during the disease in about 70% of cases, usually in the relapsing-remitting phase [113]. Meanwhile, although there are no clear survey statistics, ON is not uncommon as one of the core symptoms of NMOSD. ON causes damage to the retina and optic nerve in patients with MS and NMOSD [114, 115], and has a direct effect on the retinal structures and microvasculature, leading to vision loss and visual impairment. Thus, it is likely that retinal changes in MS and NMOSD patients are influenced by a combination of factors.

**Figure 5.**
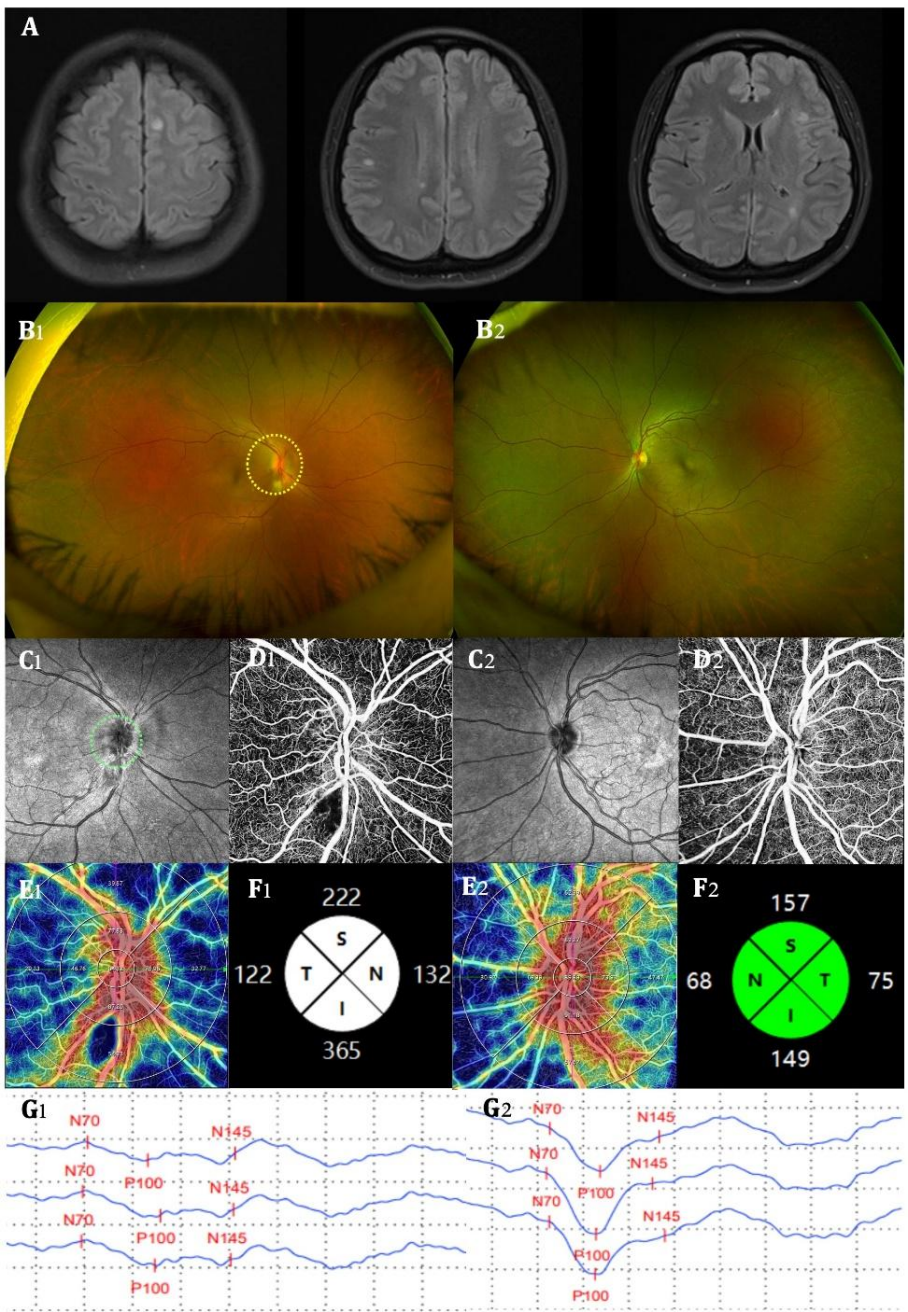
**An example of OCTA findings in a MS patient**. A 24-year-old female was admitted to the hospital due to “blurred right eye vision for 4 days accompanied by eye rotation pain”. The brain MRI T2-FLAIR sequence (A) showed multiple hyperintensities. Positive oligoclonal bands were found in CSF. Ultimately, the patient was diagnosed as MS. The fundus photography revealed that the right eye’s optic disc (yellow circle) was pale and edematous, with unclear boundaries compared with the left eye (B1 *vs.* B2). OCTA examination further revealed that the right eye exhibited disc edema (green circle) with indistinct boundaries (C1 *vs.* C2), unclear vascular boundaries (D1 *vs.* D2), decreased SRCP VD (E1 *vs.* E2), and increased pRNFL thickness due to edema (F1 *vs.* F2) compared with the left eye. The VEP test showed a decrease in the amplitude of the right eye compared to the left eye (G1 *vs.* G2). MRI, magnetic resonance imaging; CSF, cerebrospinal fluid; MS multiple sclerosis; OCTA, optical coherence tomography angiography; pRNFL, peripapillary retinal nerve fiber layer; SRCP, superficial retinal capillary plexus; VD, vessel density; VEP, visual evoked potential. N, nasal; T, temporal; S, superior; I, inferior.

### 5.2 OCTA in MS

### 5.2.1 Comparison of MS and HC

Retinal thickness was significantly thinner in MS patients compared to HC (RNFL [116-125], GCC [119-121, 124, 125], GCL [116, 119], center macular thickness [126]). The retinal vascular system (SRCP [106, 116, 119, 121, 125, 127-132], DRCP [106, 116, 119, 123, 125, 129], RPC [116, 118, 122, 124, 127, 129, 131], and peripapillary capillaries [120, 122, 130]) and the choroidal vascular system [125, 133] were also significantly impaired. The FAZ was enlarged in area [117, 121, 123, 126] and reduced in its circularity[123]. As can be seen, the fundus changes in MS patients are very significant, with extensive retinal and choroidal involvement.

### 5.2.2 OCTA parameters associated with MS diseases duration and severity

**Figure 6.**
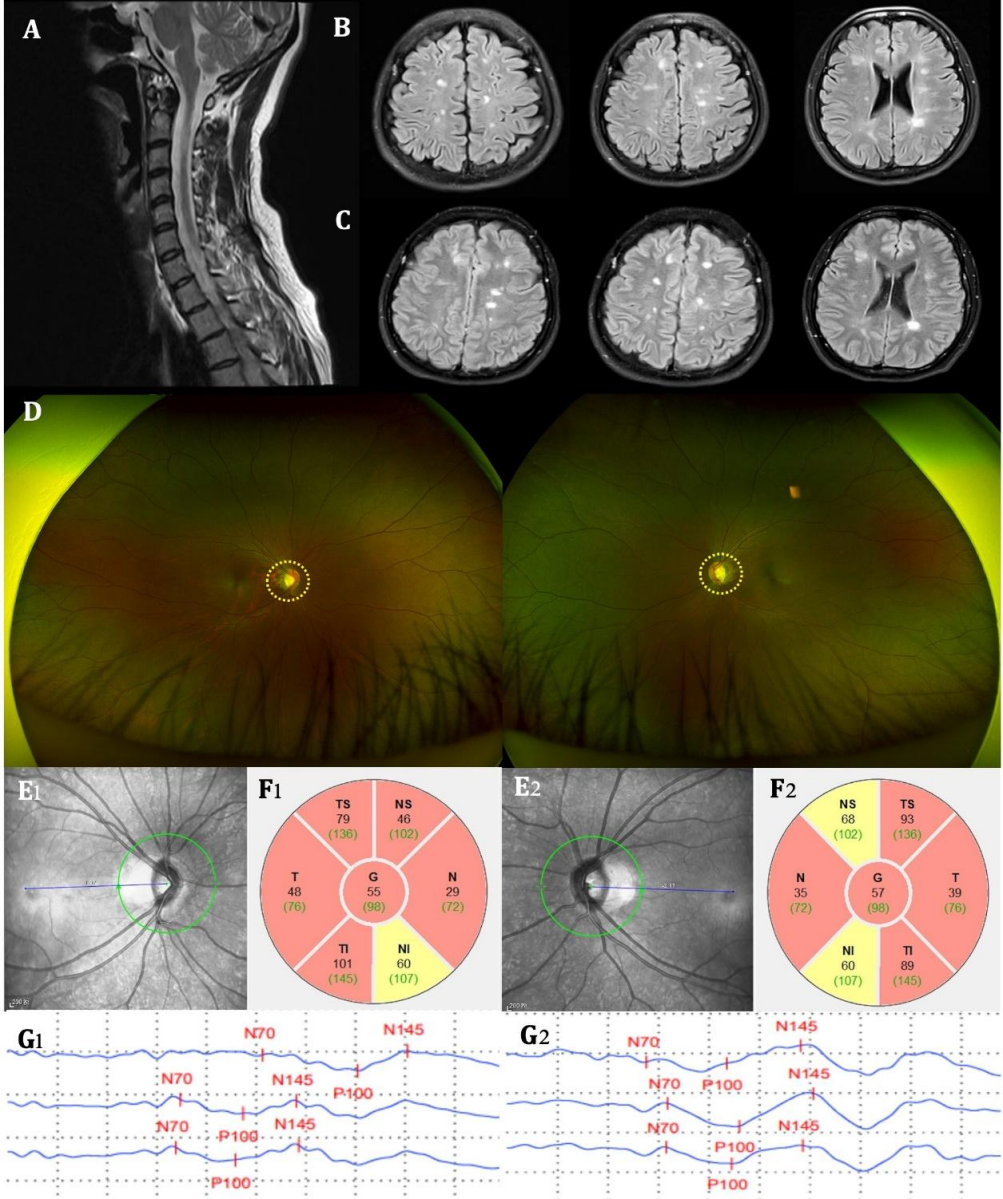
**An example of OCT findings in a RRMS patient**. A 33-year-old female was admitted to the hospital due to “recurrent limb weakness and numbness for 11 years, worsened for 2 days”. Previously diagnosed with RRMS in an external hospital. Baseline (September 27^th^, 2022) cervical (T2 sequence, A) and brain MRI (T2-FLAIR sequence, B) both found multiple hyperintensities. Positive oligoclonal bands were found in CSF. Four months later (December 13^th^, 2022), the follow-up brain MRI (T2-FLAIR sequence, C) showed a change in the number of hyperintensities. During this follow-up process, the patient's fundus photography revealed a pale color of the optic discs in both eyes (yellow circles, D). OCT also confirmed optic disc atrophy (green circles, E1-2) and reduced pRNFL thickness (black numbers, F1-2) in both eyes. VEP test showed prolonged latency and reduced amplitude of binocular recorded waves (G1-2). These fundus imaging findings indicate that this RRMS patient has experienced pronounced optic nerve damage as a result of recurrent episodes of disease remission. RRMS, Relapsing-remitting multiple sclerosis; MRI, magnetic resonance imaging; CSF, cerebrospinal fluid; OCT, optical coherence tomography; pRNFL, peripapillary retinal nerve fiber layer; VEP, visual evoked potential. G, general; N, nasal; NS nasal superior; NI nasal inferior; T, temporal; TS temporal superior; TI temporal inferior.

The researchers also found that RNFL thickness [124, 129], GCC thickness [124, 132], central macular thickness [129], DRCP [134], and RPC [124] were associated with disease progression and severity (EDSS score, MSFC score, MSSS score) in MS patients. SRCP was associated not only with the disease severity [128, 132, 134] but also with the disease duration [128]. The fundus conditions of two MS patients with different disease lengths are shown in Figures 5 and 6. The MS patients with longer disease duration had greater optic nerve damage.

### 5.2.3 OCTA parameters and CSF inflammatory factors in MS patients

A previous study by Knier et al. using OCT showed that loss of GC-IPL is associated with intrathecal B-cell immunity and constitutes an independent risk factor for worsening disability, whereas high INL volumes are associated with activity on MRI in the brain parenchyma [135]. Another study by Krajnc et al. using OCT showed that increased leukocyte counts in CSF appeared to be associated with loss of RNFL thickness [136].

In a study using OCTA to explore CSF inflammatory factors and retinal vascular biomarkers in patients with MS, Noll et al. [134] found that rarefication of the SRCP occurred during relapsing-remitting multiple sclerosis (RRMS) and was linked to higher frequencies of activated B cells and higher levels of the pro-inflammatory cytokines interferon-γ, tumor necrosis factor-α and interleukin-17 in the CSF. During a median follow-up of 23 months, vessel loss within the SRCP and DRCP was associated with future disability worsening.

The researchers hypothesized that the retinal biological parameters of MS patients are in part related to the pro-inflammatory intrathecal immunophenotype and that the changes in the retinal vascular system of MS patients may be the result of the inflammatory changes in the retina caused by MS. At the same time, retinal biological parameters may also reflect the progression and the severity of MS.

### 5.2.4 MS ON+ *vs.* HC

Compared with HC, patients with MS ON+ (eyes with an ON history) have significantly thinner retinal thickness (RNFL [116-118, 120], GCC [120], GCL [116]) and an impaired retinal vascular system [116-118, 120, 132]. In a study of MS patients with long-term follow-up, a reduction in SRCP after ON in MS patients was accompanied by thinning of the retinal GCC and GC-IPL layers. As time progressed, the loss of SRCP vessels increased and the FAZ area expanded. The investigators concluded that the impairment of SRCP after ON may be closely related to GC-IPL atrophy, which may be due to altered local metabolic activity in the inner retinal layers [137].

### 5.2.5 MS ON- *vs.* HC

Some studies have shown no significant differences between MS ON- (eyes without an ON history) and HC [124, 129, 130]. However, others have found significant changes in the fundus of MS ON- patients. For example, RNFL [117, 119, 123, 125], GCC [119], and GCL [119] were thinner, SRCP [119, 132] and DRCP [119] VD were reduced, and VDI of superficial and deep capillaries around the optic disc area was lower [120]. The choroidal vascular system is impaired [133]. FAZ area is enlarged and circularity is reduced [123]. Some studies have further explored the effect of ON attack on the fundus in MS patients. Patients with MS ON+ were found to have reduced RNFL and GCC thickness, decreased perfusion in the optic disc area[120], and reduced macular SRCP VD compared to those with MS ON- [128]. However, at the same time, other studies did not find significant changes between them.

The presumed pathophysiology of ON is inflammation and demyelination of the optic nerve[138, 139]. Activated peripheral T cells migrate across the blood-brain barrier (BBB) and release cytokines and other inflammatory mediators leading to neuronal cell death and axonal degeneration [140]. Several studies in MS have demonstrated that inflammatory demyelination is a pathological hallmark of the disease [141, 142]. A previous study found that the majority of patients with ON (74%) developed thinning of the RNFL, and it tended to occur within 3 to 6 months after the onset of ON [143]. The inflammatory factors that lead to ON in MS patients may also further disrupt the BBB in patients, thereby damaging the retinal microvascular system. The retinal structure and vascular system of some MS ON+ patients are significantly different compared with MS ON- patients. However, some studies have shown no significant change in RNFL in ON patients. Thus, we hypothesize that the damage of ON to the retinal structure of patients is not absolute, and that some of the retinal damage in MS ON+ patients may not be very severe, thus not producing a significant difference when compared with MS ON- patients. In addition, the degree of retinal involvement in MS patients varies depending on a number of factors, such as repeated remission episodes, duration of disease, and number of ON attacks. Therefore, there are differences in the findings of different MS ON- patients compared to HC and MS ON+ patients compared to MS ON- patients in different studies.

### 5.3 OCTA in NMOSD

### 5.3.1 NMOSD *vs.* HC

Retinal thickness was significantly thinner in NMOSD patients compared to HC (RNFL [116-118, 144-148], GCL [116, 144, 147], central macular thickness and volume [117, 144, 149, 150], ONL [144], INL[144]). The retinal vascular system (SRCP [116, 144, 147, 150-153], ICP [150], DRCP [116, 144, 150-153], RPC [116, 118, 144, 145, 147, 148], and peripapillary capillaries [117, 146]) was impaired, and the FAZ was enlarged [149, 150].

### 5.3.2 OCTA parameters associate with NMOSD diseases duration and severity

**Figure 7.**
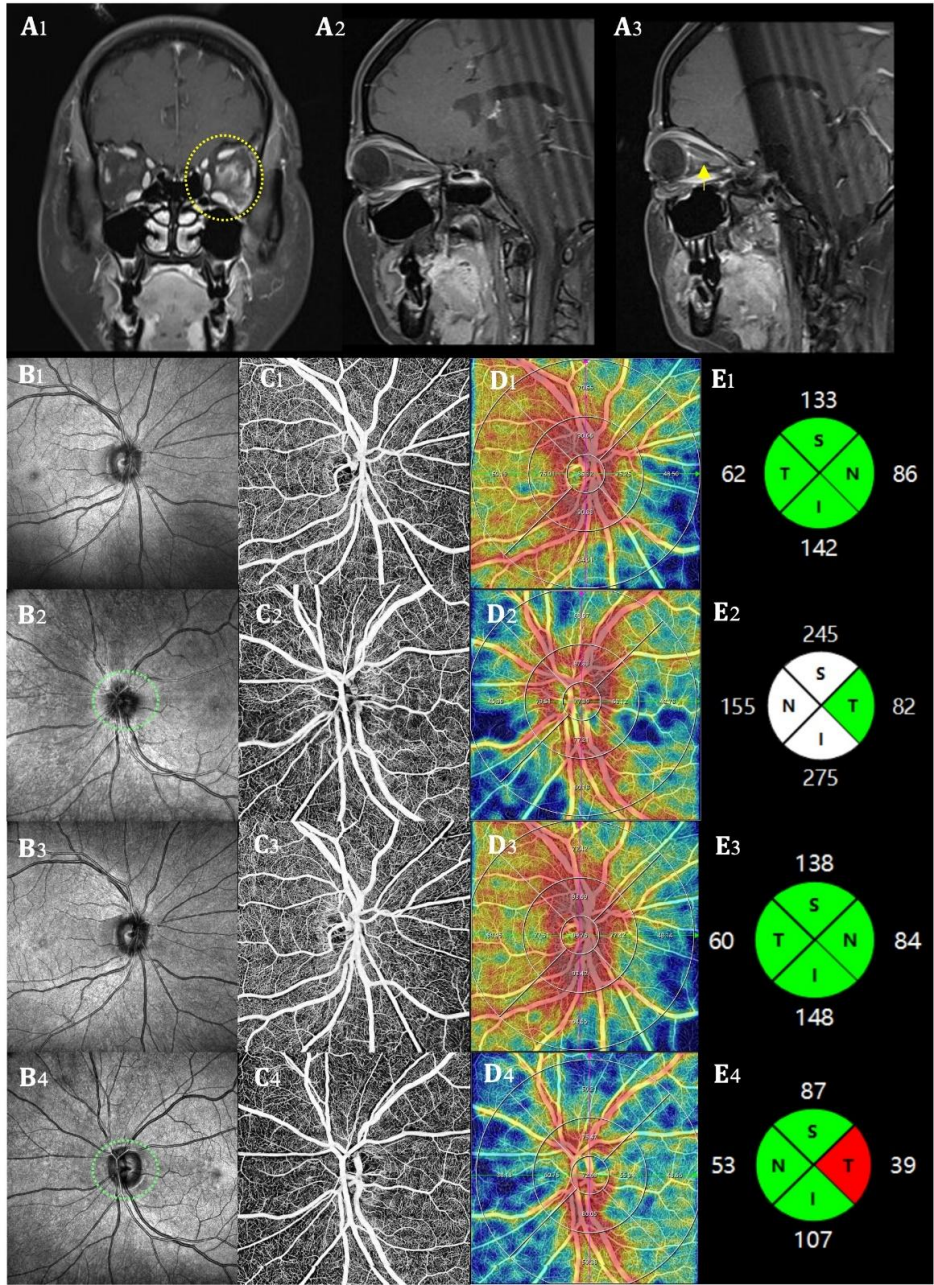
**An example of OCTA findings in a NMOSD patient**. A 21-year-old male was admitted to the hospital due to “left eye vision decreased for 4 days”. Baseline (September 4^th^, 2023) Orbital MRI (Coronal position, A1) showed a patchy enhancement in the left eye (yellow circle). MRI T1-contrast sequence showed no obvious abnormality in the right eye optic nerve (A2), but an abnormal enhancement in the left eye optic nerve (yellow arrow, A3). CSF APQ4 antibody test was positive. OCTA examination revealed that, compared with the right eye, the optic disc of the left eye was pale, edematous, and had unclear borders (B2 *vs.* B1). Additionally, in the left eye, the vascular border was unclear (C2 *vs*. C1), the SRCP VD was decreased (D2 *vs*. D1), and the pRNFL thickness was increased due to edema (E2 *vs*. E1). Four months later (December 7^th^, 2023), the symptoms of optic neuritis in the left eye had improved, with the edema subsiding compared to the acute phase. The four-month follow-up OCTA examination showed normal findings in the right eye (B3-E3). In the left eye, the optic disc border (B4 *vs*. B2) and the vascular border also became clearer than baseline (C4 *vs*. C2), however, there was a further decrease in SRCP VD (D4 *vs*. D2) and pRNFL thickness (E4 *vs*. E2), particularly in the temporal region (red fan-shaped area, E4). This suggests that patients with NMOSD may still experience persistent damage to the fundus vessels and optic nerve after the resolution of optic neuritis. MRI, magnetic resonance imaging; NMOSD, neuromyelitis optica spectrum disease; OCTA, optical coherence tomography angiography; SRCP, superficial retinal capillary plexus; VD, vessel density; pRNFL, peripapillary retinal nerve fiber layer; N, nasal; T, temporal; S, superior; I, inferior.

Researchers have found that retinal thickness (RNFL, GCL [150]) and vascular parameters in patients with NMOSD correlate with the duration of NMOSD[150, 152], disease severity [150, 153], and the number of ON attacks [144, 150, 152]. The fundus conditions of a patient with NMOSD examined by OCTA at different times during the onset of ON and after the ON had resolved are shown in Figure 7.

### 5.3.3 NMOSD ON+ *vs.* HC

When conducting subgroup analysis on NMOSD patients, researchers found that RNFL thickness [116-118, 146-148], GCL thickness [116, 147], and central macular thickness[117, 150] decreased in NMOSD ON+ patients. SRCP [116, 117, 147, 150-153], ICP [150], DRCP [116, 150-153], and RPC[116, 147, 148] are damaged. Peripapillary VD is also damaged [117, 146]. The FAZ area is enlarged [150]. Tiftikcioglu et al. also found that the cup-to-disc ratio and cup volume of the optic disc in NMOSD patients who have experienced ON were significantly increased compared to HC [117].

### 5.3.4 NMOSD ON- *vs.* HC

Impairments in retinal thickness [150, 152] and the macular microvasculature [117, 148, 150-153] were also found in NMOSD ON- patients. This may be caused by pathologic changes in the visual cortex and other parts of the brain in NMOSD patients [154]. Huang et al. found that retinal VD had decreased before ON occurred and RNFL atrophy occurred, which represents a possible subclinical primary retinal vasculopathy in NMOSD [148]. In addition, some studies have confirmed that there is extensive lesion of cerebral white matter in NMOSD [155]. A study by Ma et al. addressing the correlation between brain function and biological parameters of retinal vascular in patients with NMOSD found an association between both the pRNFL thickness and RPC VD and the functional connectivity changes in the brain respectively. RPC VD showed a significant association between the right lingual gyrus, bilateral calcarine gyrus, and left thalamus, respectively. The pRNFL thickness showed significant association with the right lingual gyrus, right calcarine gyrus, and left thalamus respectively [156]. Meanwhile, another study by Wang et al. also found a negative correlation between RNFL thickness and RPC VD and functional brain connectivity in NMOSD patients [145]. All of the above studies confirm that structural and microvascular damage around the optic disc is closely linked to changes in brain function in NMOSD.

### 5.4 Changes in OCTA after treatment for MS and NMOSD

Fingolimod is a first-in-class sphingosine 1-phospate (S1P) receptor modulator therapy to be approved for the treatment of RRMS. It is an effective drug that significantly decreases the number of relapses in RRMS patients, slows disability progression, reduces inflammatory activity, and lowers the cerebral volume loss rate in MRI lesions. Previous studies using OCT and OCTA to observe retinal changes in RRMS patients taking fingolimod have shown conflicting results on macular and choroidal thickness changes after fingolimod use. Kal et al. showed that patients who had used fingolimod for 12 months had higher choroidal thickness at 1500 μm temporal and nasal to the fovea, and 1000 μm nasal to the fovea, than patients with MS who had not been treated with the drug [157]. Kal et al. suggested that this is due to the therapeutic effect of fingolimod in MS patients. Fingolimod is a structural analog of S1P. The S1P receptor plays a role in regulating vascular permeability and enhancing endothelial barrier integrity. Fingolimod can inhibit this barrier action and may lead to increased vascular permeability. However, a study by Karaküçük et al. using OCTA found that choroidal thickness was significantly lower in MS patients who had been taking fingolimod for more than 6 months than in those who had been taking it for less than 6 months [126]. Karaküçük et al. concluded that the mechanism of fingolimod's effect on the choroid remains unclear. The conflicting results of choroidal thickness changes in MS patients treated with fingolimod may be due to the fact that the choroidal blood supply is not homogeneous, and differences in localized changes in blood flow have led to different results between studies.

Karaküçük et al. also found that MS patients who used fingolimod for more than 6 months had a significant reduction in central macular thickness compared with those who used it for less than 6 months. However, the study by Nolan et al. found that MS patients who did not use fingolimod showed a significant reduction in macular volume at an average 12-month follow-up compared with HC [158]. Fruschelli et al. found no significant changes in central macular thickness or total volume at 12-month follow-up in RRMS patients treated with fingolimod [159]. Al Rashdi et al. found that during the 4-year follow-up of RRMS patients receiving fingolimod treatment, there was no significant change in macular center thickness compared to baseline data at the beginning of treatment, but there was a decrease in macular volume towards the end of the study [160]. However, Nørgaard et al. reported an increase in macular volume and thickness in 138 MS patients taking fingolimod at three to four months follow-up [161].

We believe that the lower central macular thickness in MS patients taking fingolimod with a follow-up period greater than 6 months may be due to the natural progression of RRMS. In a North American Fingolimod Phase 3 trial in RRMS, a two-year follow-up showed a reduction in mean RNFL in 34.0% of patients, and macular thickness in 25.0% of patients [162]. Fingolimod causes retinal macular edema in some patients with MS, increasing macular thickness and volume. The side effects usually occur within 3 months of starting fingolimod treatment and gradually subside after cessation of the treatment [163]. Few studies have used OCTA to observe retinal microvascular changes in MS patients after fingolimod treatment. Karaküçük et al. found that SRCP perfusion was significantly higher in patients who had been using fingolimod for less than 6 months than in patients who had been using it for more than 6 months and in HC. It may be because fingolimod can block S1P1 and S1P3 receptors in early administration, thus downregulating adhesion complexes and increasing retinal vascular permeability.

However, there are rare studies using OCTA to observe retinal changes before and after NMOSD medication use until now, which awaits future exploration.

### 5.5 Differences in OCTA findings between MS and NMOSD patients

Previous studies using OCT to evaluate MS and NMOSD patients concluded that NOMSD ON+ patients had more significant RNFL and GCL thinning compared to MS ON+ patients [164-167]. This was further confirmed in studies using OCTA [116, 117, 127]. In addition, researchers found that NMOSD ON+ patients also had significantly thinner macular volumes [117] and reduced SRCP [116] and RPC VD [116-118, 127]compared to MS ON+ patients. There were also significant differences in retinal parameters between NMOSD patients and MS patients, even without episodes of ON. NMOSD ON- patients had a significantly higher RPC VD [118, 127] and a smaller FAZ area[117] compared with MS ON- patients.

The damage to the optic nerve and retinal structures is more severe in NMOSD compared to MS. Several previous studies using OCT have confirmed this finding [164, 166, 168-170]. We also found that vascular impairment was generally more severe in NMOSD patients. This may be due to several factors: (1) axonal damage is more severe in NMSOD than in MS, which leads to a greater reduction in retinal oxygen and nutrient requirements, resulting in a greater decline in retinal perfusion in NMOSD than in MS patients; (2) The main clinical feature of NMOSD is predominant axonal damage within the anterior visual pathway, in contrast to MS, where demyelination spreads along the entire visual pathway [171]. This may lead to differences in the main areas of damage and the severity of optic nerve damage between the two diseases, resulting in differences in the patient's retinal structure, further leading to differences in the degree of secondary retinal vascular changes in each disease; (3) The difference in retinal VD between the two diseases may be due to different pathophysiologic mechanisms that trigger the primary vasculopathy; (4) NMOSD results in the loss of perivascular GFAP-positive astrocytes [172], whereas previous studies have found that GFAP is generally upregulated in MS [173]. GFAP is a cytoskeletal protein related to the structure and motility of astrocytes. Astrocytes are known to bridge neuronal activity to vascular responses [174]. Patients with NMOSD are at high risk for neurovascular coupling damage caused by astrocyte dysfunction, resulting in more severe damage to retinal structures and the vascular system than in MS.

## 6 Cerebrovascular disease

### 6.1 CAS

CAS is a disease that threatens human health, with age, hyperlipidemia, smoking, coronary artery disease and so on being its main risk factors [175]. CAS is a major cause of cerebral microcirculatory dysfunction [176, 177] and accounts for 20% of ischemic strokes [178, 179]. 10-15% of all new strokes are due to untreated CAS [180]. CAS can lead to death and permanent disability [180, 181]. In addition, CAS leads to decreased cerebral perfusion and impaired microcirculatory function, which can further trigger the development of cerebral small vessel diseases (CSVD) and promote microangiopathy, white matter lesions, and lacunar infarction [182-186], ultimately leading to vascular dementia [187]. Timely diagnosis and treatment of CAS are crucial in reducing the incidence of ischemic stroke and its complications. Currently, Doppler ultrasound is the preferred diagnostic method for CAS due to its convenience and speed, but human factors during the examination may lead to increased measurement variability and error. CT or MR angiography is a more objective method, mainly used for revascularization, but is more expensive and invasive. However, none of these methods can observe damage to the cerebral microvasculature.

Previous studies have generally concluded that CAS can lead to decreased retinal blood flow because of (1) Anatomy. Carotid stenosis (>80%) may result in a significant decrease in blood pressure in the ophthalmic vascular bed [188]. The ophthalmic artery is the first intradural branch of the internal carotid artery, and stenosis or occlusion of the carotid artery is likely to result in inadequate perfusion and impaired circulation in the eye on the same side of the stenosis, or even in the contralateral eye, with consequent reduction in the blood flow to the central retinal artery and the CC, and apoptosis of the retinal ganglion cells, cone cells and rod cells [189]. Even mild CAS has been reported to be strongly associated with ocular ischemic syndrome (OIS). Depending on the severity and duration of the reduction in blood flow, this can lead to the development of a variety of ocular disorders such as transient monocular vision loss (transient blackout), OIS, central retinal artery embolism, and glaucoma in patients with CAS. Ultimately, permanent vision loss or even blindness results. Additionally, the length and shape of carotid atherosclerotic plaques can also have an impact on retinocervical blood flow [190]; (2) Low cranial pressure caused by carotid artery stenosis. When the carotid artery is severely narrowed (>70%) or occluded, eye blood flow often branches into the low-resistance intracranial circuit (ophthalmic artery steal phenomenon), leading to a decrease in retrobulbar blood flow and affecting retinal blood flow perfusion [191-193]; (3) Serotonin release from atherosclerotic plaques. In some patients with atherosclerosis, serotonin released by platelet aggregation on atherosclerotic plaques may trigger vasoconstriction of vessels in the optic nerve head and/or retina and thus contribute to their ischemia disorders. This transient constriction and spasm of the ophthalmic arteries may play a role in the development of dark haze or even permanent ocular ischemic lesions, resulting in a greater reduction in flow than that caused by arterial stenosis alone [194]; (4) Other factors. CAS patients are often accompanied by other systemic vascular diseases, such as hypertension, hyperlipidemia, diabetes, etc., all of which may affect eye blood flow and lead to subclinical changes [195].

However, not all CAS can cause a significant decrease in retinal blood flow. This may be due to: (1) Narrow site. Sometimes the ophthalmic artery originating from an atherosclerotic internal carotid artery has a markedly stenosed lumen at its origin from the internal carotid artery, with little or no stenosis of the internal carotid artery itself [188]; (2) Collateral circulation and self-regulatory capacity of the carotid and ophthalmic arteries. The presence of collateral circulation and self-regulatory capacity can play a compensatory role so that retinal blood flow does not undergo significant perfusion reduction in patients with mild to moderate carotid stenosis (<50%). Even, in some patients with severe carotid artery stenosis or occlusion, the contralateral eye may not experience significant blood flow changes and may not develop OIS [196, 197]; (3) Medication. Most patients with CAS take multiple medications such as statins, anti-hypertensives, and anticoagulants. These drugs can promote and increase the blood perfusion to the retina and form a certain degree of improvement to the retinal blood supply [198, 199].

### 6.1.1 CAS ipsilateral eyes *vs*. HC

Previous studies using OCTA in patients with CAS have shown contradictory results regarding choroidal thickness compared to HC. Some studies have shown that in CAS patients, the choroid thickness becomes thinner [195, 200, 201] and the choroidal vascular is impaired [201, 202]. Researchers have suggested that the reduced choroidal thickness in the CAS ipsilateral eye may be related to reduced blood flow in the carotid artery, which leads to lower blood pressure, reduced choroidal blood flow, and ultimately to choroidal thinning. However, Rabina et al. found no significant difference in choroidal thickness in patients with CAS compared with HC [203]. Additionally, Monferrer-Adsuara et al. found that patients with severe CAS have significantly higher middle and deep choroidal VD than HC and VD increases with the degree of CAS [204]. The reason for the contradictory results of choroidal thickness changes may be that the choroidal blood circulation is irregular [205], resulting in localized changes in choroidal blood flow caused by CAS. Reduced ocular blood flow may also disturb vascular conformation and resistance, resulting in disruptions in the choroid following multiple compensation mechanisms such as collateral circulation or the presence of ‘steal phenomenon’ [191]. The primary role of the choroid is to nourish the outer retina, and the increase in choroidal VD may be a compensatory response by the eye to provide more blood to the ischemic retina. This has led to inconsistent findings in numerous small-sample cross-sectional studies.

The retinal structure and vascular system of CAS patients were found to have reduced VD and PFD in the SRCP [195, 200, 206-209], DRCP [195, 206-208, 210], RPC [195, 207, 208, 210], and overall vascularization of the macular region [211] compared to HC. The FAZ circularity was reduced [200]. Among them, the SRCP showed the most prominent performance. This may be due to the fact that compared to the deep retinal vessels, the SRCP is more superficial, more finely vascularized, and more susceptible to injury.

### 6.1.2 CAS ipsilateral eyes *vs*. contralateral eye

Previous studies have suggested that because of collateral circulation and self-regulation of the carotid and ophthalmic arteries, the contralateral vessel can compensate and maintain a normal blood supply even if one side of the vessel is severely stenotic or occluded. Some OCTA studies have also found significant differences in retinal structure and blood supply between the CAS ipsilateral eye and the contralateral eye. GCL [202], RNFL [202] and choroidal layer became thinning [195, 201, 212]. SRCP [195, 208, 213], DRCP [208, 210, 213], RPC [195, 210], and peripapillary optic disc vasculature [209, 210] were impaired. The choroidal vascular system was also impaired [201, 202], And VD was reduced in the FAZ region [195].

### 6.1.3 Comparisons of OCTA parameters in ipsilateral eyes among patients with different degrees of CAS

A subset of studies grouped CAS patients according to the degree of stenosis and performed subgroup analyses. The study by Liu et al. found reduced SRCP VD and choroidal thickness in CAS patients with <50% stenosis compared to HC, and in CAS patients with >50% stenosis compared to CAS patients with <50% stenosis. There was a significant difference in RPC VD between mildly stenotic and non-mildly stenotic CAS patients. However, DRCP VD, was not found to be significantly different between the groups [200]. On the other hand, Li et al. found significant reductions in SRCP, DRCP, and RPC VD in CAS patients with 50%-70% stenosis versus HC, as well as stenosis >70% versus CAS patients with 50%-70% stenosis[208]. Monferrer-Adsuara et al. found that CAS patients with stenosis >70% and stenosis of 30-70% had increased mid-choroidal VD compared to patients with CAS <30% stenosis. CAS patients with >70% stenosis had increased deep choroidal VD compared to patients with <70% stenosis [204].

### 6.1.4 Associations between OCTA parameters and carotid plaque and cerebral perfusion markers in CAS patients

Studies by Cao et al. that used OCTA to observe the correlation between retinal vascular parameters and indicators of carotid stenosis in CAS patients found that retinal microvascular changes (SRCP and DRCP VD) are related to the degree and length of the stenosis [206, 213]. The correlation between the degree of stenosis and retinal blood flow may be due to the decrease in carotid blood flow as the stenosis becomes more severe, resulting in reduced retinal blood flow. On the other hand, the correlation between stenosis length and retinal blood flow may be due to the fact that patients with longer stenoses have an increased risk of dislodging atherosclerotic debris or thrombus and are more susceptible to embolization. In addition, carotid stenosis length may also have a significant effect on internal carotid artery collateral flow and cerebral artery blood flow [214]. In a study by Xu et al, SRCP and DRCP were negatively correlated with carotid intima media thickness and common carotid artery plaque [206].

There is also an association between retinal biological markers and changes in cerebral perfusion in patients with CAS. Magyar-Stang et al. found that retinal VD was linked to cerebral arterial resistance-transient hyperemic response ratio [202, 211, 215]. Liu et al. found that SRCP correlated with relative cerebral blood volume (rCBV), relative cerebral perfusion (rCBF), and relative cerebral permeability(rPS) [202, 211, 215]. Meanwhile, Kwapong et al. found that RNFL, GC-IPL thickness, and CVI were also associated with rCBV and rPS [202, 211, 215]. We can speculate that retinal vascular perfusion is related to the impairment of cerebral hemodynamics. This indicates a correlation between the carotid artery and cerebral blood flow, which are biological parameters of the retina in CAS patients. The use of OCTA to detect fundus vasculature in CAS patients for early diagnosis of the disease shows promise in preventing disease progression.

**Figure 8.**
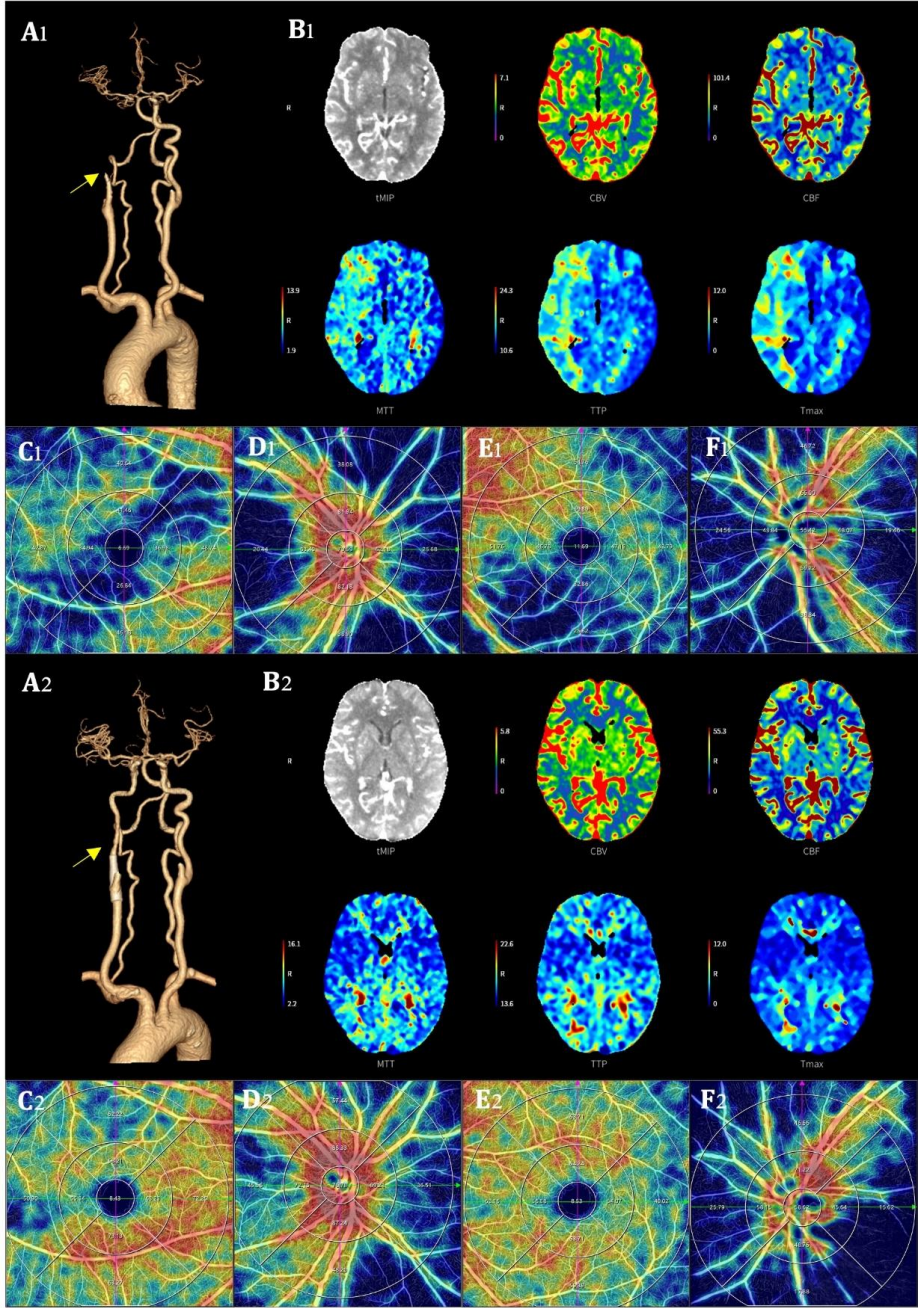
**An example of OCTA findings in a CAS patient**. A 66-year-old male accepted carotid artery stent surgery due to severe stenosis of the right carotid artery (>90%). Baseline (December 27^th^, 2023) CTA and CTP showed an occlusion of the right ICA (C2-C7 segments) (yellow arrow, A1), and extensive hypoperfusion in the right hemisphere (B1). In his pre-operative OCTA images, the SRCP VD in the macular (C1, E1) and optic disc (D1, F1) regions of both eyes were significantly reduced, especially seen in the right eye (C1*vs*. D1, E1*vs*. F1). Three days after the surgery (January 12^th^, 2024), the follow-up CTA image revealed that the occluded right ICA was successfully recanalized (yellow arrow, A2 *vs*. A1), and the baseline intracranial hypoperfusion was reversed to normal (B2 *vs*. B1). The post-operative OCTA images showed significant improvement of the SRCP VD of the macular (C2 *vs.* C1, E2 *vs.* E1) and optic disc areas (D2 *vs.* D1, F2 *vs.* F1) in his both eyes, indicating that the blood flow perfusion of the retina in both eyes has been restored. CAS, carotid artery stenosis; CTA, computer tomography angiography; CTP, computer tomography perfusion; ICA, internal carotid artery; OCTA, optical coherence tomography angiography; SRCP, superficial retinal capillary plexus; VD, vessel density.

### 6.1.5 Changes in OCTA parameters before and after carotid stenting or carotid endarterectomy

Surgical treatment for CAS mainly consists of carotid stenting and carotid endarterectomy (CEA). Some investigators used OCTA to observe changes in fundus vascular parameters in CAS patients before and after surgical treatment. Compared with the baseline data of CAS patients before surgical treatment, RNFL thickness[208], choroidal thickness [212, 216], and retinal vascular parameters (SRCP [208, 217], DRCP [208, 217], RPC [208, 217], and peripapillary blood vessels of the optic disc region [208, 217]) were significantly restored in CAS patients within only 1 week after the surgery. A study of CAS patients with a 3-month follow-up after surgery found that CAS patients experienced an improvement in VD in the ipsilateral eye. At the same time, retinal perfusion improved significantly in both the ipsilateral and contralateral eyes [210]. This suggests that carotid stenosis surgery not only contributes significantly to the restoration of carotid blood flow, but also to the improvement of retinal perfusion. However, in a 3-month follow-up of CAS patients (>70% stenosis) after surgical treatment, Machalińska et al. found a decrease in the central retinal venular equivalent (CRVE) in both the ipsilateral and the contralateral eye after CEA[218]. A decrease in the central retinal arteriolar equivalent (CRAE) was also found in the ipsilateral eye. The researchers concluded that despite the restoration of carotid blood flow after surgery, retinal microvascular dysfunction is likely to be long-lasting. The reason for the differing results of these two studies may be that some patients had more severe CAS for a longer period of time, resulting in greater damage to the retinal microvasculature and slower, lesser recovery after surgery. However, it remains to be seen if researchers will conduct large sample-size studies with more long-term follow-up for CAS patients undergoing surgery for CAS. An example of one CAS patient examined by OCTA before and after the carotid artery stent surgery is shown in Figure 8.

### 6.2 Ischemic stroke

Stroke is one of the major causes of disability and death worldwide. Various classifications exist based on criteria such as ischemic/hemorrhagic and brain site [219]. The major risk factors for ischemic and hemorrhagic stroke are hypertension, dyslipidemia, and diabetes [220-222]. 87% of all strokes are ischemic (obstruction of blood flow to the brain) and the remaining 13% involve hemorrhagic events (rupture of blood vessels in the brain) [223]. Ischemic stroke is the result of most systemic diseases involving abnormalities of the large blood vessels and microvessels, leading to reduced blood flow and injury in specific brain areas.

Previous studies using fundus photography to explore retinal changes in stroke patients concluded that the retinal vascular FD is generally reduced in patients who have had a stroke [224-230]. This condition may be due to two factors: (1) Decreased blood supply. Stroke occurs with regional damage to the brain, decreased cerebral perfusion, and cerebral microvascular dysfunction [231], which leads to decreased blood supply to the retina. The retina has a high demand for oxygen and blood, and ischemia and hypoxia result in damage to the retinal vascular system, which leads to a decline in the retinal vascular FD; (2) Trans-synaptic degeneration. After ischemic stroke, with regional damage to the brain, trans-synaptic degeneration may be seen in post-synaptic neurons (anterograde) and pre-synaptic neurons (retrograde). In the eye, retrograde trans-synaptic degeneration takes place in the retinal ganglion cells that project to the lateral geniculate nucleus after the death of the neurons that synapse with these cells, ultimately leading to thinning of the retinal ganglion cells [232, 233] and RNFL [234]. In turn, the thinning of retinal structures leads to a reduced demand for oxygen and blood flow to the retina, which results in an impaired retinal blood flow system. In addition, studies using OCT have found that ischemic stroke patients have reduced choroidal thickness under the central macular [235]. The choroid plexus of the brain is a capillary complex that acts as a barrier against harmful substances and secretes a variety of growth factors that contribute to brain development. The choroid is likewise a vascular structure that serves as a barrier against harmful toxins and contributes to the secretion of various growth factors that contribute to retinal development[236]. In animal models, ischemic stroke has been shown to cause ischemic necrosis and cellular damage in the choroid plexus [237].

### 6.2.1 Ischemic stroke patient *vs*. HC

**Figure 9.**
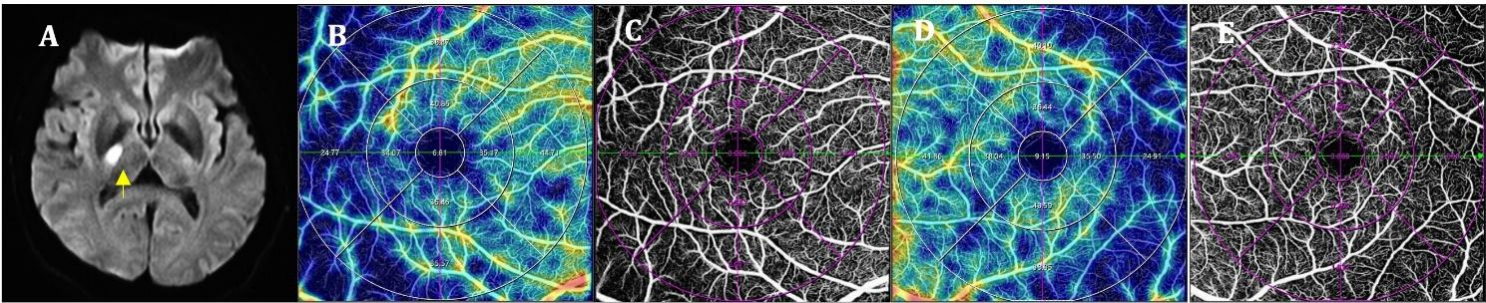
**An example of OCTA findings in an ischemic stroke patient**. A 50-year-old male was admitted to the hospital on February 1^st^, 2022, due to an infarction in the right internal capsule (yellow arrow, A). OCTA examination (one year after stroke onset) was conducted and revealed that the right eye exhibited a decrease in SRCP VD (B *vs.* D) and PA (C *vs.* E) compared with the left eye. MRI, magnetic resonance imaging; OCTA, optical coherence tomography angiography; SRCP, superficial retinal capillary plexus; VD, vessel density; PA, perfusion area.

A study by Molero-Senosiain et al. using OCTA to observe retinal structural and vascular parameters in stroke patients showed a thinning of the macular and the GCL thickness in ischemic stroke patients compared to HC. Additionally, DRCP VD and vascular area (VA) was found to be reduced, along with a decrease in the outer retina layer VD. The total of RPC, SCP, and ICP VA was reduced[238]. Liang et al. found that GCC and RNFL thickness were reduced, and SRCP, DRCP, and RPC VD were generally lower in ischemic stroke patients compared to HC [239]. Liu et al. found that after correcting for the participants' gender, visual acuity, systolic and diastolic blood pressures, smoking history, hemoglobin (HbA1c), cholesterol, and high-density lipoprotein (HDL) levels, VD remained lower in all subregions of the SRCP and DRCP in stroke patients. The VD around the FAZ and the VD of the optic disk were also lower than HC[240]. The results may indicate that the reduction in retinal VD in stroke patients is not related to traditional risk factors for stroke, which makes the use of retinal microvascular parameters to monitor stroke more convincing. In a study by Duan et al, logistic regression results showed that higher FAZ axis ratio and lower FAZ circularity in DCP were associated with ischemic stroke [241]. In addition, Pachade et al. using OCTA to observe the retina of patients in the acute phase of ischemic stroke found a significant reduction in SRCP, and DRCP VD of acute stroke patients compared to HC [35]. It is evident that reduced macular microvessel density may be associated with acute stroke to some extent [242]. An example of one ischemic stroke patient examined by OCTA is shown in Figure 9.

### 6.2.2 Lacunar cerebral infarction patient *vs.* HC

Lacunar stroke, also known as recent single subcortical infarction (RSSI), accounts for a quarter of all ischemic strokes[243]. RSSI is usually caused by CSVD or steno-occlusion of a parent artery in the brain [244, 245]. Previous studies using fundus photography have found that a narrower central retinal arteriole equivalent, wider central retinal venule equivalent, focal arteriolar narrowing, and arteriovenous nicking were predictive of lacunar stroke. Retinal imaging is useful in understanding the pathophysiology and mechanisms of lacunar stroke [246, 247].

A recent study by Kwapong et al. using OCTA to observe retinal changes in patients with RSSI [248] found that the retina microvessels of RSSI patients were significantly sparser, and the choroid was thinner compared to HC. The change in retina microvessels and choroid were both significantly correlated with MoCA scores. CSVD load and NIHSS scores were also significantly correlated with retinal microvessels in RSSI patients. A study by Cao et al. found [249] that RSSI patients had thinner RNFL thickness and sparser SRCP, DRCP, and RPC VD than HC.

### 6.3 Migraine

Migraine is a complex multifactorial neurologic disorder. It is characterized by attacks of unilateral, throbbing head pain, with sensitivity to movement, visual, auditory, and other afferent inputs [250]. It is widely recognized by researchers today that the trigeminal vascular pain pathway is a common pathway that leads to the development of migraine. The trigeminovascular system (TGVS) consists of the trigeminal nucleus, the trigeminal ganglion, the trigeminal nerve, and its innervated meningeal and ocular vascular networks. The activation of the TGVS increases the release of neurotransmitters and vasoactive intestinal peptides, which activate injurious receptors that transmit pain signals to the center via the trigeminal nociceptive afferent fibers, resulting in pain. During visual aura or migraine attacks, reduced blood flow due to transient vasospasm can cause perfusion deficits, occurring not only in the cranial but also in the ocular vasculature. Migraineurs may have acute and chronic changes in ocular perfusion, and recurrent migraines can also cause permanent damage to the brain and retina [251, 252]. It has been reported that migraine is closely associated with various neurovascular eye diseases such as retinal artery occlusion, ischemic optic neuropathy, and glaucoma [253-255].

A previous case report by González-Martín-Moro et al. found [256] that OCTA scan images of a 27-year-old male migraine patient during an acute attack showed a large hypoperfused area in the macular region of the right eye. After 48 hours, along with the improvement of the patient's visual symptoms, the OCTA showed a significant reduction in the area of hypoperfusion. Seven days later the patient was asymptomatic and retinal perfusion had returned to normal values. In a case report by Bingöl Kızıltunç et al [257], a 34-year-old woman with a history of migraine with aura three times in one year was relieved with naproxen sodium. OCTA images of the patient's right eye at the onset of visual aura showed diffuse stenosis of the retinal vessels in the right eye, with decreased SRCP, DRCP, and RPC VD. However, all of these changes recovered 3 hours after the visual aura. Based on these results, it is hypothesized that migraine with visual aura has some effects on the retinal vascular system during the attack, but these effects can be completely resolved over time.

However, along with a growing number of published studies, researchers have identified cortical spreading depression (CSD) as a major mechanism in the development of migraine aura, which can lead to synaptic activity, changes in extracellular ion concentrations, and the release of calcitonin gene-related peptide (CGRP) from trigeminal nerve endings. CGRP, as the main driver of neurogenic meningeal vasodilation in migraine, eventually leads to an increase in regional cerebral blood flow, known as spreading congestion, which lasts approximately 1-2 minutes, followed by prolonged hypoperfusion lasting 1-2 hours. These changes in cerebral blood flow and oxygenation lead to further aggravation of the development of ischemia and subsequent parenchymal lesions [258], triggering secondary vasoconstriction and ischemia in the retinal vasculature.

Previous studies using OCT to observe retinal and choroidal thickness in patients with chronic migraine have found that migraine with or without aura cause varying degrees of impairment to the retinal structure and vascular system. The RNFL [50-53] and choroidal thickness [51, 54] are significantly thinner in migraine patients compared to HC. A study using OCTA in migraine patients also found that choroidal thickness in migraine patients correlated with the disease duration.

Studies using OCTA to observe migraine patients have similarly found that RNFL thickness [259-261] is significantly lower in migraine patients compared to HC, consistent with previous OCT studies. It may be due to the lack of blood supply to the retina and optic nerve as a result of vasospasm and retrobulbar blood reflux caused by migraine attacks. On the other hand, the RNFL consists mainly of nerve fibers composed of retinal ganglion cell axons, which are mostly unmyelinated require more energy supply, and are therefore more susceptible to retinal ischemic damage [262]. However, in a study of pediatric migraine, it was shown that the macular central fovea thickness was significantly higher in pediatric migraineurs than in HC [263]. Researchers assumed that the macular fovea, especially its superficial capillaries may be more sensitive to blood circulation changes than in adults. The elevated foveal retinal thickness may be a compensatory mechanism.

Using OCTA to observe retinal and choroidal blood flow parameters in migraine patients, researchers have mostly focused on exploring SRCP VD [259, 261, 263-266], DRCP VD [261, 264, 266-268], and FAZ area [259, 264, 266, 268-270]. It is currently believed that retinal perfusion is significantly reduced and FAZ area is enlarged in migraine patients compared to HC. In addition, some studies have also found significant decreases in optic nerve VD [261, 265, 266, 268] and RPC VD [260, 268]. These results demonstrate that migraine affects both the macular and optic disc regions, with comprehensive and widespread involvement of fundus blood flow, making retinal capillaries ischemic and hypoxic, ultimately resulting in decreased retinal VD and blood flow. Subsequently, in a subgroup analysis of migraine patients, the reduction in SRCP and DRCP VD and the enlargement of FAZ area were particularly significant in migraine patients with aura [259, 261, 265, 266, 268-270], whereas the differences were not significant in migraine patients without aura compared with HC [260, 263-265, 267]. Differences in ocular blood flow parameters between migraineurs with and without aura were also not significant, with previous positive findings only in SRCP VD [259] and choroidal capillary [270, 271]. This may be because poor perfusion of the posterior occipital lobes of the cerebral hemispheres is more pronounced in patients with aura than in patients without aura [272], resulting in a more pronounced restriction of blood flow to the eye in the former, leading to more severe damage to the retina. Also, basilar artery vascular reactivity was significantly reduced in patients with migraine with aura, indicating an impairment in the adaptive cerebral hemodynamic mechanisms in the posterior circulation in patients with migraine with aura [273]. However, this cannot rule out the influence of factors such as limited inclusion of cases and selection bias in the aforementioned studies. In addition, previous studies have found that macular and peripapillary capillary density and perfusion, as well as FAZ area, are significantly correlated with disease duration [269, 274], frequency of migraine attacks [265, 266], and migraine severity (e.g., migraine disability assessment questionnaire, MIDAS score, and HIT score) [264-266] in migraine patients.

**Figure 10.**
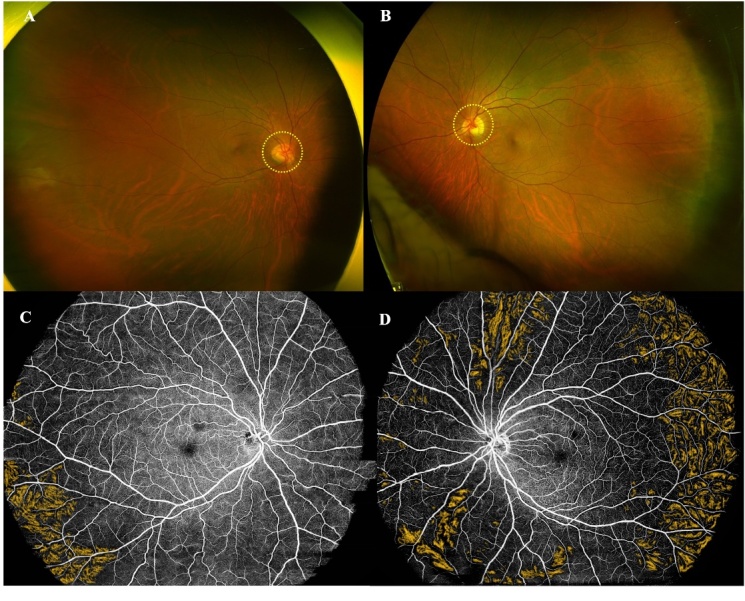
**An example of OCTA findings in a migraine patient**. A 51-year-old female admitted to our hospital due to “recurrent headache for about 40 years”. This patient experienced 3-5 times migraine attacks per year since the beginning of menstruation, without receiving any medication treatment. Upon ophthalmic examination, her best-corrected visual acuity was found to be 1.0 in the right eye and 0.4 in the left eye. The fundus photography revealed normal findings in the right eye, while the optic disc in the left eye appeared pale compared to the right eye (yellow circles, B *vs.* A). The OCTA 26 × 21mm scanning image showed a larger non-perfused area in the left eye fundus and a lower vascular density than the right eye (orange area, D *vs.* C). OCTA, optical coherence tomography angiography.

In summary, ocular blood flow is significantly lower in migraine patients compared to HC. Previously, Kara et al. also observed lower blood flow in the central retinal artery and posterior ciliary artery in migraine patients than in HC during an attack using color Doppler ultrasound [275], confirming that a migraine attack restricts ocular retinal and choroidal blood flow, which leads to a certain degree of retinal and choroidal damage. An example of one migraine patient examined by OCTA is shown in Figure 10.

## 7. Rare disease

### 7.1 LHON

LHON is a maternally inherited mitochondrial DNA-related disease. The majority of patients with LHON (90-95%) harbor one of three primary mtDNA point mutations: m.3460G>A, m.11778G>A, and m.14484T >C. LHON is remarkable in its tissue selectivity since this disorder is mainly limited to damaging retinal ganglion cells and their axons in the optic nerve. In the early stages of this disorder, the smaller-caliber fibers of the papillomacular bundle are principally lost. Later, the fiber loss may progress and eventually involve the whole optic nerve [276]. Clinical symptoms of LHON generally manifest as acute, painless loss of central vision. Severe visual impairments are often difficult to recover from. The likelihood of visual recovery is greatest with the m.14484T>C mutation, and least with the m.11778G>A mutation, with the m.3460G>A mutation having an intermediate visual prognosis. In the acute phase, LHON displays several typical clinical features, such as vascular tortuosity of the central retinal vessels, swelling of the RNFL, and peripapillary capillary dilatation microangiopathy [277], which occurs mainly in the inferior and temporal quadrants of the optic disc. Retinal microangiopathy accumulates as LHON enters the chronic phase. Within six weeks, optic nerve pallor becomes apparent, initially more marked temporally due to early axonal loss within the papillomacular bundle. Pathological cupping of the optic disc can occur with more extensive loss of retinal ganglion cells axons [278].

### 7.1.1 Case reports of LHON patients

In a previous case report of a patient with LHON using OCTA, Takayama et al. reported a 5-year-old male patient with parapapillary telangiectatic blood vessels in both eyes [279]. Gaier et al. reported a case of a man in his 30s with a maternal family history of LHON and a known m.11778G>A mutation presented with 10 days of subacute, painless vision loss in the left eye [280]. OCTA confirmed the presence of dilated peripapillary microvasculature that was most prominent temporally in the inner retinal layers of the left eye. Similar changes were noted in the right eye, also in a temporal distribution. In a case report by De Rojas et al., a 17-year-old man had acute vision loss in the left eye, mitochondrial DNA testing revealed a T14484C mutation, suggestive of LHON [281]. Acutely, there is hyperemia of the optic nerve, circumpapillary microangiopathy, and dilated, tortuous vasculature. In a case report by Asanad et al. in a patient with LHON with the m.3460G>A mutation, the patient had RNFL pseudo edema in both eyes and thinning of the GCC. Temporal microvascular detachment was noted in the optic papillae and peripapillary region in both eyes. In addition, vascular attenuation was seen on the nasal side of the peripapillary region. In a report by Bingöl Kızıltunç et al [282], an LHON male with the G11778A mutation had thinning of the whole quadrant of the RNFL in the right eye, decreased VD in the whole quadrant of the RPC, and vascular detachment. The left eye had thinning of the inferior and temporal quadrants of the RNFL and reduced RPC VD with vessel detachment in the inferior and temporal quadrants. Progressive decrease in RNFL and RPC VD over 12 months of follow-up was observed.

The above case reports reflect that both the optic disc and peripapillary capillaries were damaged to some extent in patients with LHON.

### 7.1.2 Comparisons of OCTA parameters between LHON patients *vs.* HC

In a previous study using OCTA, Kousal et al. [283] examined 5 eyes of 6 LHON patients with different mutation loci and found thinning of the RNFL, especially in the temporal region. As well as a decrease in RPC VD. In the GCL layer, there was a significant difference between only two individuals with severe symptoms and normal human controls. In addition, no significant differences were found between any of the unaffected eyes of LHON carriers and normal individuals. In contrast, a study by Borrelli et al. [284] found that 29 eyes of 15 LHON patients had significantly thinner GCC and RNFL, and significantly lower parafovea VLD in SRCP and DRCP, compared to 20 eyes of 20 HC. In particular, SRCP and DRCP VLD were lower in the nasal and inferior regions, and DRCP PFD was lower in the nasal region.

### 7.1.3 Changes of OCTA parameters in different stages of LHON

In the study by Balducci et al [285], which used OCTA to observe different progression stages of LHON, twenty-two LHON patients were divided into four groups: unaffected mutation carriers (LHON-u) (n=8), early subacute stage (LHON-e) (n=4), late subacute stage (LHON-l) (n=5) and chronic stage (LHON-ch) (n=9). A significant decrease in temporal RPC VD was detected in LHON-u patients. In LHON-e, the VD was reduced in the temporal and inferior-temporal sectors compared with controls. In LHON-l, VD was reduced in whole, temporal, superior-temporal, and inferior-temporal sectors compared with LHON-u and controls. In LHON-ch, the VD was reduced in all sectors compared to the other groups. An asynchronous pattern emerged in the temporal sector with VD changes occurring earlier than RNFL thickness changes and together with GC-IPL thinning. The above results indicate that RPC vascular lesions appear early in the course of LHON and progressively increase in extent and severity as the disease progresses. RPC parameters have the potential to be useful biomarkers for monitoring the disease process of LHON, evaluating therapeutic efficacy, and elucidating pathophysiology.

Castillo et al. also used OCTA to observe the fundus of LHON patients at different times (n=60) [286]. The SRCP, DRCP, and RPC vessels were all found to undergo significant vascular attenuation in LHON patients compared to HC, which was further exacerbated with disease progression. The SRCP was the only vessel that already showed significant impairment in the asymptomatic phase of LHON disease. Vascular changes were first detected in the SRCP in areas of the papillomacular bundle, the region preferentially affected by LHON disease. However, the temporal sector of the RPC showed the greatest magnitude of change as LHON progressed. Thus, the RPC was shown to be ultimately more severely impaired during the LHON-ch phase.

### 7.2 ALS

ALS is the most common adult-onset neurodegenerative disease of the motor system [287], with the average age of onset is 60 years [288]. ALS is characterized by the combined degeneration of both upper motor neurons located in the motor cortex and lower motor neurons located in the spinal cord. The progressive degenerative process affects both upper and lower motor neurons leading to progressive paralysis. Death occurs within three to five years of diagnosis, mainly due to respiratory failure. The pathophysiologic mechanisms by which neurodegeneration occurs in ALS are still unclear [289-292], and researchers are currently proposing a variety of hypotheses, including microvascular damage in CNS. This hypothesis is supported by several elements such as the pathogenicity of mutations in the vascular endothelial growth factor (VEGF) and angiogenin genes, and the presence of neuronal intracytoplasmic inclusions of TDP-43 in models of hypoperfusion and cerebral ischemia [293].

OCTA studies for ALS are still limited, with only one study conducted by Cennamo et al [294]. The study included 48 ALS patients and 45 HC. Cennamo et al. categorized the ALS patients according to the rate of disease progression into the slow progression group (n=10), intermediate progression group (n=24), and fast progression group (n=14). The examination did not show any significant difference in GCC and RNFL thickness between the three ALS subgroups and normal controls. However, the subfoveal choroidal thickness (SFCT) was significantly increased in ALS patients and was thicker in patients with slow and moderate disease progression than in those with fast disease progression. Moreover, the Amyotrophic Lateral Sclerosis Functional Rating Scale Revised score (ALSFRS-r) correlated with SFCT in ALS patients. However, OCTA vascular parameters did not show any significant results. Future studies should focus on conducting long-term follow-up studies with larger sample sizes of ALS patients.

### 7.3 WS

WS is a rare autosomal recessive progressive disease [295] caused largely by mutations in the WFS1 gene and [296, 297], less commonly, mutations in the CISD2 gene (WS type 2). WS is characterized by childhood onset insulin-dependent diabetes mellitus and optic atrophy associated in a variable number of cases to diabetes insipidus, sensorineural hearing loss and neurodegeneration [298, 299]. The prognosis for WS is poor, with most patients dying from severe neurologic deficits. WFS1 gene encodes for wolframine, a transmembraneous protein in the endoplasmic reticulum (ER) and is highly expressed in brain tissue, pancreatic β-cells, and in the heart. Of note, loss of function mutations of WFS1 gene triggers a cascade of ER and mitochondrial dysfunction that ultimately leads to apoptosis and cellular death [300, 301]. Wolframin is also expressed in retina, specifically in retinal RGC and in non-myelinated portion of the optic nerve [302, 303].

The only OCTA-related study on WS is in a retrospective study by Battista et al [304], which included 10 WS patients, with a disease duration range from 4 to 29 years, 8 eyes of type 1 diabetic patients and 17 HC. All participants were imaged by OCT and OCTA. The results of the study showed that the mean RNFL and GCC thickness were significantly lower in WS patients, PFD was significantly lower in SRCP, DRCP and ONH, and VLD was smaller in SRCP and ONH compared to diabetic patients and HC. However, the VLD was increased in DRCP compared with type 1 diabetic patients. Battista et al. analyzed that this may be because the significant impairment of the RNFL and GCC in WS patients may result from a series of ER and mitochondrial dysfunctions triggered by mutations in the WFS1 gene, which ultimately lead to optic nerve cell apoptosis and cell death. Following severe damage to the RNFL and GCC, a reduction in retinal energy demand, a secondary reduction in vascular flow, and a loss of vascular support structures lead to secondary damage to the retinal microvasculature. However, given the scarcity of OCTA studies on WS, more in-depth exploration by researchers is still needed in the future.

## 8. The advantages and limitations of OCTA in CNS studies

Advantages of OCTA over other fundus imaging techniques include: (1) Non-invasive, rapid and economical assessment. Both brain MRI and OCTA are non-invasive, while the latter one has the advantages in rapid assessment and cost-effective, which has great potential in broadscale screening of CNS disease patients in future; (2) Multilayered analysis. Compared with fundus photography, OCTA can longitudinally tomograph the retina, revealing the multilayered structure of the retina in real time. It is therefore capable of reflecting microvasculature lesions in different layers of retina and choroidal capillaries in CNS disease patients; (3) Accessible to blood flow. Compared with OCT, OCTA can visualize blood flow in vessels down to the capillary level, which enables researchers to observe retinal microvascular changes in patients with CNS disease in a more in-depth and detailed manner and accurately assess the degree of microvascular damage; (4) Real-time quantification. Measurement software that equipped on OCTA not only automatically segments the retinal layers, but also directly obtains a variety of retina-related quantifiable parameters, making it more convenient, objective, and reproductive than before to evaluate the retinal structure and blood flow changes in CNS disease patients.

The limitations of OCTA are reflected in: (1) the heterogeneity of measurement. It is caused by different machine algorithms and product models. Since the introduction of OCTA by the US FDA in 2015, OCTA machines have undergone continuous updates and rapid development. Differences in machine algorithms and product models have resulted in varying precision and accuracy of measurement results between OCTAs; (2) Requirement of high-degree cooperation from patients. An important limitation of OCTA is that the examination requires a high degree of patient cooperation and attention. However, many neurodegenerative diseases reduce the ability of understanding and self-control in CNS disease patients, resulting in motion artifacts and poor imaging quality in OCTA images, leading to inaccurate analysis of the results; (3) Comorbidities interfere with OCTA image quality. Patients with neurodegenerative diseases are usually elderly, and most of them have ocular comorbidities such as hypertensive retinopathy, diabetic retinopathy, retinal vein occlusion, and age-related macular degeneration. Previous studies have found a significant overlap between fundus changes associated with retinal vascular disease and retinal changes caused by neurodegenerative diseases. Therefore, it is extremely challenging to accurately characterize the extent to which each disease affects the retinal vasculature. In the same context, other macular or optic neuropathies, such as the preretinal membrane or myelinated nerve fiber layer, may also blur the images obtained by the OCTA device; (4) the limited scanning range. The scanning range of OCTA is still narrow compared to wide-angle fundus photography, and even with commercial systems gradually starting to use larger 12 × 12mm scanning modes, it still does not allow for a wider exploration of the peripheral field of fundus lesions in CNS disease. The eye lesions caused by CNS diseases can be randomly distributed in any area of the fundus. However, due to the limitations of current OCTA technology, the scanning field is mostly limited to the posterior pole of the retina, which cannot fully cover the peripheral retina. These limitations of OCTA are highly likely to lead to missed diagnosis of eye lesions in quite a number of CNS disease patients.

## 9. Prospects

OCTA can provide 3D vascular images, which indicates that powerful post-processing software or artificial intelligence (AI) will hold great promise in the future in the field of rapid recognition and image analysis of CNS disease. It is of great practical significance to utilize AI technology to identify the characteristic changes of CNS disease on OCTA, promoting the early screening of CNS disease in the future.

To date, there are few studies using OCTA to explore the correlation between choroidal parameters and CNS disease. The choroidal microcirculation accounts for more than 80% of the blood flow to the eye, and the choroid consists of Bruch’s membrane, CC layer, Sattler’s layer and Haller’s layer [305]. Due to signal attenuation caused by light scattering from the RPE layer and CC layer, the vascular layer blood flow in the Sattler’s and Haller’s layers is unable to displayed very clearly on OCTA. Besides, researchers still have not precisely delineated the boundaries of the Sattler’s and Haller’s vascular layers. Therefore, studies on choroidal layers on OCTA still mainly focus on the CC until now. However, with the advancement of OCTA analyzing techniques, the blood flow changes in the deeper layers of the retina as well as the choroidal layer can be observed more accurately and meticulously than in the past, and this type of study is also expected.

Through the summary of our review, it can be found that in previous OCTA studies of CNS disease patients, there have been some clear OCTA parameters, such as the VD of SRCP and DRCP. In the future, by integrating these indicators, it is possible to establish predictive models related to CNS disease by integrating these indicators.

## Conclusion

OCTA may be the future “Tower of Babel” for visualizing CNS disease. To date, numerous studies using OCTA have detected a variety of retinal microvascular abnormalities in patients with CNS disease. However, there is a lack of definitive evidence on retinal biomarkers in the diagnosis of CNS disease and the changes in retinal biomarkers that occur as CNS disease progresses. Most of the published OCTA studies also suffer from a number of methodological limitations, mainly on sample sizes, study design, data collection protocols, and analytic methods. Therefore, future standardized methods for retinal assessment and CNS disease characterization, along with larger sample-sized prospective cohort studies are needed to determine the potential value of OCTA in future clinical practice.

## Data Availability

The materials generated during the present review are available from the corresponding author on reasonable request. Considerations will be made based on the reasons for requesting the materials and the procedures for ensuring data privacy.
